# Non-Steroidal Anti-Inflammatory Drugs Increase Cisplatin, Paclitaxel, and Doxorubicin Efficacy against Human Cervix Cancer Cells

**DOI:** 10.3390/ph13120463

**Published:** 2020-12-15

**Authors:** Diana Xochiquetzal Robledo-Cadena, Juan Carlos Gallardo-Pérez, Víctor Dávila-Borja, Silvia Cecilia Pacheco-Velázquez, Javier Alejandro Belmont-Díaz, Stephen John Ralph, Betsy Alejandra Blanco-Carpintero, Rafael Moreno-Sánchez, Sara Rodríguez-Enríquez

**Affiliations:** 1 Departamento de Bioquímica, Instituto Nacional de Cardiología, Mexico City 14080, Mexico; xochiquetzal@ciencias.unam.mx (D.X.R.-C.); jcga_1999@yahoo.com.mx (J.C.G.-P.); suerte11@hotmail.com (S.C.P.-V.); belmont81@hotmail.com (J.A.B.-D.); betsyblanco9519@gmail.com (B.A.B.-C.); rafael.moreno@cardiologia.org.mx (R.M.-S.); 2 Laboratorio de Oncología Experimental, Instituto Nacional de Pediatría, Mexico City 14080, Mexico; latrans86@hotmail.com; 3 Menzies Health Institute Queensland, School of Medical Science, Griffith University, Gold Coast, QLD 4222, Australia; s.ralph@griffith.edu.au

**Keywords:** Bliss-type additivism model, celecoxib, dimethylcelecoxib, drug synergism, HeLa cells, resistance index

## Abstract

This study shows that the non-steroidal anti-inflammatory drug (NSAID) celecoxib and its non-cyclooxygenase-2 (COX2) analogue dimethylcelecoxib (DMC) exert a potent inhibitory effect on the growth of human cervix HeLa multi-cellular tumor spheroids (MCTS) when added either at the beginning (“preventive protocol”; IC_50_ = 1 ± 0.3 nM for celecoxib and 10 ± 2 nM for DMC) or after spheroid formation (“curative protocol”; IC_50_ = 7.5 ± 2 µM for celecoxib and 32 ± 10 µM for DMC). These NSAID IC_50_ values were significantly lower than those attained in bidimensional HeLa cells (IC_50_ = 55 ± 9 µM celecoxib and 48 ± 2 µM DMC) and bidimensional non-cancer cell cultures (3T3 fibroblasts and MCF-10A mammary gland cells with IC_50_ from 69 to >100 µM, after 24 h). The copper-based drug casiopeina II-gly showed similar potency against HeLa MCTS. Synergism analysis showed that celecoxib, DMC, and casiopeinaII-gly at sub-IC_50_ doses increased the potency of cisplatin, paclitaxel, and doxorubicin to hinder HeLa cell proliferation through a significant abolishment of oxidative phosphorylation in bidimensional cultures, with no apparent effect on non-cancer cells (therapeutic index >3.6). Similar results were attained with bidimensional human cervix cancer SiHa and human glioblastoma U373 cell cultures. In HeLa MCTS, celecoxib, DMC and casiopeina II-gly increased cisplatin toxicity by 41–85%. These observations indicated that celecoxib and DMC used as adjuvant therapy in combination with canonical anti-cancer drugs may provide more effective alternatives for cancer treatment.

## 1. Introduction

Quality of life for most cancer-bearing patients becomes severely compromised after chemo- and/or radiotherapy, due to the numerous side effects (hematological, gastrointestinal, hepatic, and/or renal damages) associated with these aggressive treatments [1]. To minimize these unwanted adverse effects, several approved clinical protocols that include combining two or more drugs are under development, with the rationale of improving their efficacy whilst decreasing toxic side effects from each drug used alone. It is realized that many clinical trials have applied two or more drugs which, when used together, have been directed to the same target or to different proteins within the same biological process/function [2,3,4], but they have yielded poor outcomes [5,6]. Hence, novel treatment strategies are urgently needed, which may achieve greater responses with decreased tumor progression and lower adverse side effects.

To prevent the harmful complications from anti-cancer drug-related side effects, novel chemotherapy strategies may be based on the simultaneous use of multiple drugs with combined greater efficacy but given at comparatively lower doses. The aim is to target two or more different essential cancer cell processes such as cell proliferation, signaling, and/or metabolism. In this regard, the repurposing of approved drugs could be a promising alternative anti-cancer strategy. The reason is that the careful selection of such drugs may have advantages in that (i) they should show less side effects; (ii) they may have unconventional but effective targets in tumor cells; and (iii) their use may decrease the overall cost and time associated with the development of brand-new chemotherapeutics [7,8].

Celecoxib is a non-steroidal anti-inflammatory drug (NSAID) that is widely used for the treatment of pain, fever, inflammation, and rheumatic diseases [9]. In addition to its anti-inflammatory activity [10], it has been shown that celecoxib, at micromolar doses, also decreases the fast growth of high metastatic breast and colorectal (MDA-MB-231, MDA-MB-468, Caco-2, SW-480, HT-29) and low metastatic breast (MCF-7) cancer cells [11,12,13], with a limited effect on non-cancer cells [12,13].

The anti-cancer effects of celecoxib are associated with the following: (i) the inhibition of the highly over-expressed tumor cyclooxygenase-2 (COX-2), which in turn has been related to the acquisition and maintenance of an invasive metastatic phenotype [14]; (ii) the activation of the intrinsic apoptosis pathway [15]; (iii) the inhibition of oxidative phosphorylation (OxPhos) [12,13]; and (iv) the blocking of cell migration and invasiveness [12]. The combination of celecoxib with some drugs (PD184161, ZD6474, or plumbagin) has been already successfully tested on several carcinomas (gallbladder cancer, osteosarcoma, melanoma) [16,17,18]. However, some of these latter drugs are currently still under development, and they have not yet been approved by the USA Food and Drug Administration, which will most likely delay their eventual entry into the clinic [19,20]. In contrast, celecoxib has already been tested, in combination with several commonly used and approved chemotherapy drugs (temozolomide, cisplatin, gemcitabine, and etoposide), on glioblastoma, pancreatic, and small-cell lung cancer [21,22]. Unfortunately, for all of these particular combinations, the overall response to restrain cancer progression has been rather modest (6–11 months), and side effects including dyspepsia, diarrhea, and abdominal pain were frequently observed [21,22,23]. Thus, the search for better combinations with celecoxib, or other NSAIDs, appears clinically relevant for improving current treatments.

The celecoxib analogue, 2,5-dimethylcelecoxib (DMC) has also shown potent anti-cancer effects against several malignant carcinomas (Burkitt’s lymphoma, myeloma, non-small cell lung, gastric, breast, and colon carcinomas) [24,25,26,27,28,29]. Unlike celecoxib, DMC lacks COX-2-inhibitory function [27,30]. Thus, the COX-2-inhibitory function seems not to be required for the anti-proliferative and anti-tumorigenic properties of these NSAIDs. To date, scarce studies have been reported analyzing the synergistic effect of celecoxib or DMC with canonical anti-cancer drugs in patients with cervix cancer [31].

Therefore, the aim of the present study was to find effective combinations of celecoxib or DMC with several canonical anti-cancer drugs, particularly those used in the common clinical treatment of cervix cancer. It was thought that the combination of these drugs, at relatively lower doses, (i) could potently decrease growth of bidimensional and/or tridimensional (multi-cellular tumor spheroid model, MCTS) human cervix cancer HeLa and SiHa and human glioblastoma U373 cell cultures; whilst (ii) exhibiting minimal activity on non-cancer cells. MCTS represents a useful in vitro model for early-stage, avascular tumors as well as micrometastasis [32,33] and also for testing the ability of drugs to penetrate into tumors [34,35], resembling the in vivo situation with solid tumors.

In the present study, an extensive drug screening was undertaken to identify synergistic combinations of drugs, which target different functions in the malignant stage IV drug-resistant HeLa cervical cancer cell line [36,37], in order to provide promising chemotherapeutic alternatives to deter malignant and drug-resistant cancer types. The HeLa cell line was selected as a cancer cell model because it shows several advantages over other cancer cell lines. HeLa cells represent one of the cancer types with higher incidence in women of reproductive age and show a high metastatic degree compared to other cancer cell lines [38]. HeLa cells exhibit high resistance to different drugs used in clinical chemotherapy as compared to other highly metastatic (breast MDA-MB-231, breast MDA-MB-468) carcinomas [39]. HeLa cells also show the ability to produce uniform high-size multi-cellular tumor spheroids (MCTSs), which is crucial to obtain valid, reliable, and reproducible data for testing anti-cancer drugs and their combinations [40,41]. These features make HeLa cells a suitable model for studying multidrug-resistance mechanisms and drug synergism. For comparison, another human cervix cancer cell line (SiHa) as well as the highly metastatic human glioblastoma U373 cells were also used.

## 2. Results

### 2.1. Effect of NSAIDs and Canonical Chemotherapy Drugs on HeLa Cell Proliferation

**Figure 1:**
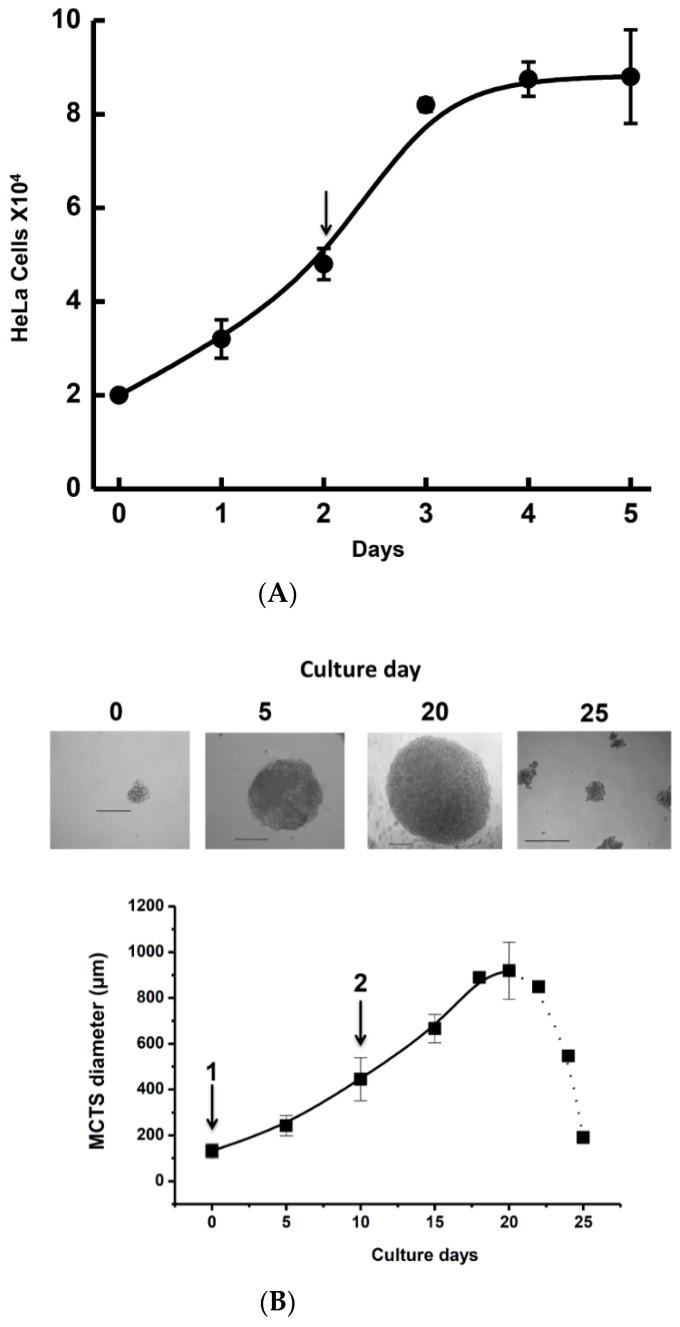
HeLa cell growth in bidimensional (**A**) and tridimensional (**B**) cultures. Arrows indicate the time points where the drug was added to the cell culture. For multi-cellular tumor spheroids (MCTS), drugs were added at the beginning of the culture for the preventive protocol (1) or once spheroids were formed for the curative protocol (2). Bar = 200 µm.

For bidimensional HeLa cell cultures (Figure 1A; doubling time of 19 ± 4 h), the IC_50_ values for each drug alone (Table 1) were determined after 24 h incubation with drug present from day 2 (as indicated by the arrow in Figure 1A) to day 3. Casiopeina II-gly (CasII-gly) was used as a non-canonical, experimental anti-cancer drug and showed greater potency (IC_50_ = 1.5 µM) for inhibiting HeLa cell proliferation compared to NSAIDs and the other canonical chemotherapy drugs (Table 1). At these doses, it has been demonstrated that CasII-gly blocks several enzymes in the Krebs cycle, thereby inhibiting OxPhos flux [42,43]. All canonical anti-cancer drugs except carboplatin (IC_50_ in the mM range) showed greater toxicity against HeLa cells than did the NSAIDs. All the IC_50_ values obtained here were within the range of values reported for the same drugs in HeLa cells [43,44,45,46].

For HeLa MCTS growth, the largest spheroid diameter (910 ± 124 µm; *n* = 30 spheroids) was reached at around day 20 (Figure 1B). After day 20, fast spheroid disintegration was usually attained. It was noted that the IC_50_ values in the preventive protocol (i.e., when the drugs were added at the beginning of MCTS formation) were one order of magnitude lower than those determined in the curative protocol (i.e., when the drugs were added once the spheroids were formed) (Table 1). Interestingly, celecoxib and DMC showed the greater toxicity on spheroid formation and growth, as compared to CasII-gly or canonical anticancer drugs. The high NSAIDs toxicity observed with MCTS using the preventive protocol was similar to that reported for LNCaP prostate MCTS incubated with experimental drugs such as MLN4924, which is a ubiquitin ligase-like protein inhibitor [47]. In contrast to what is observed in bidimensional cultures that required millimolar concentrations, carboplatin was required in nano or micromolar concentrations to block spheroid growth (Table 1).

### 2.2. Synergism of NSAIDs or CasII-Gly with Chemotherapy Anticancer Drugs

In order to evaluate whether the effects of celecoxib, DMC, or CasII-gly were synergistic when combined with chemotherapy drugs, HeLa cells in bidimensional and tridimensional cultures were treated with the canonical chemotherapy drugs at sub-IC_50_ values (Table 2, Table 3 and Table 4). For bidimensional cultures, two effects for celecoxib and DMC emerged (Table 2), as revealed by the Bliss-type additivism (BTA) analysis [48] (Figure S1). There were supra-additive or synergistic effects when the NSAIDs were combined with either cisplatin, paclitaxel, doxorubicin, or gemcitabine (15 to 79%); the stronger drug synergy was achieved when combining NSAIDs with cisplatin (Table 2).

In contrast, infra-additive effects were observed when the NSAIDs were combined with cyclophosphamide or carboplatin (−18 to −103%). The negative values indicated that the NSAIDs together with the anti-cancer drugs might have interfered with each other or with their targets, thereby diminishing their respective inhibitory effects [48]. Furthermore, combinations of NSAIDs with the vitamin E analogues α-tocopheryl succinate (α-TOS), α-tocopherol ether linked acetic acid analog (α-TEA), or methoxy-tocopheryl oxyacetic acid (M-TEA) only rendered infra-additive effects, except for celecoxib/α-TEA combination in bidimensional HeLa cell cultures (Table S1).

The resistance index (RI) (Table 3) and the combination index (CI) (Table S2) values from the drug combinations yielded similar results to those obtained with the BTA analysis in bidimensional HeLa cell cultures. The combination of celecoxib or DMC with cisplatin, paclitaxel, or doxorubicin produced RI values higher than 2 for HeLa cells, contrasting the RI values of around one for 3T3 fibroblasts; the combination of NSAIDs and canonical drugs provided CI values lower than one, indicating supra-additive effects. These observations further supported the finding that NSAIDs have synergistic effects with canonical chemotherapy drugs on the growth of cervical cancer cells. The greater RI value was attained with celecoxib *plus* cisplatin.

For tridimensional cultures, celecoxib or DMC combined with cisplatin, paclitaxel, or doxorubicin yielded BTA supra-additive effects of 12 to 76% in the preventive protocol (Table 4); DMC but not celecoxib combined with gemcitabine also showed a supra-additive effect of 25%. Both NSAIDs also exhibited supra-additive effects (7–74%) when combined with cisplatin, paclitaxel, and doxorubicin in the curative protocol (Table 4), with NSAID *plus* cisplatin showing the greater synergism; in addition, DMC also provided a supra-additive effect (11%) with carboplatin. On the contrary, marked infra-additive effects were observed in both protocols with NSAIDs combined with cyclophosphamide (Table 4). The RI values determined for HeLa MCTS concurred with the BTA data, with RI values higher than 2 for both NSAIDs combined with the chemotherapy drugs under both curative and preventive protocols (Table 3).

The combination of sub-IC_50_ CasII-gly values with the canonical chemotherapy drugs produced supra-additive effects (18–64%; Table 2) and RI values much higher than 2 (Table 3) in bidimensional cultures, whereas no infra-additive effects were observed (Table 2). Infra-additive effects in HeLa bidimensional cultures were observed when CasII-gly was combined with either α-TOS, α-TEA, or M-TEA. The BTA negative values ranged from −3 to −28%, indicating that CasII-gly was interfering with the vitamin E analogues and their anti-cancer effects (Table S1).

CasII-gly also promoted an enhanced canonical drug toxicity for HeLa MCTS under both the preventive and curative (26 to 85%) protocols, except for cyclophosphamide, which showed infra-additive values (−3 to −18%) in both protocols (Table 4). The RI values of 4–6 for the CasII-gly/cisplatin combination indicated a therapeutically attractive synergism (Table 3). Similar to what was observed in bidimensional cultures, CasII-gly combined with α-TOS or α-TEA also produced infra-additive effects in the HeLa MCTS model (Table S1), further suggesting that the use of vitamin E analogues together with CasII-gly interfered with each other and, counter-intuitively, favor the growth of cervix cancer.

### 2.3. Effect of NSAIDs on the Proliferation IC_50_ Values of Canonical Chemotherapy Drugs

**Figure 2:**
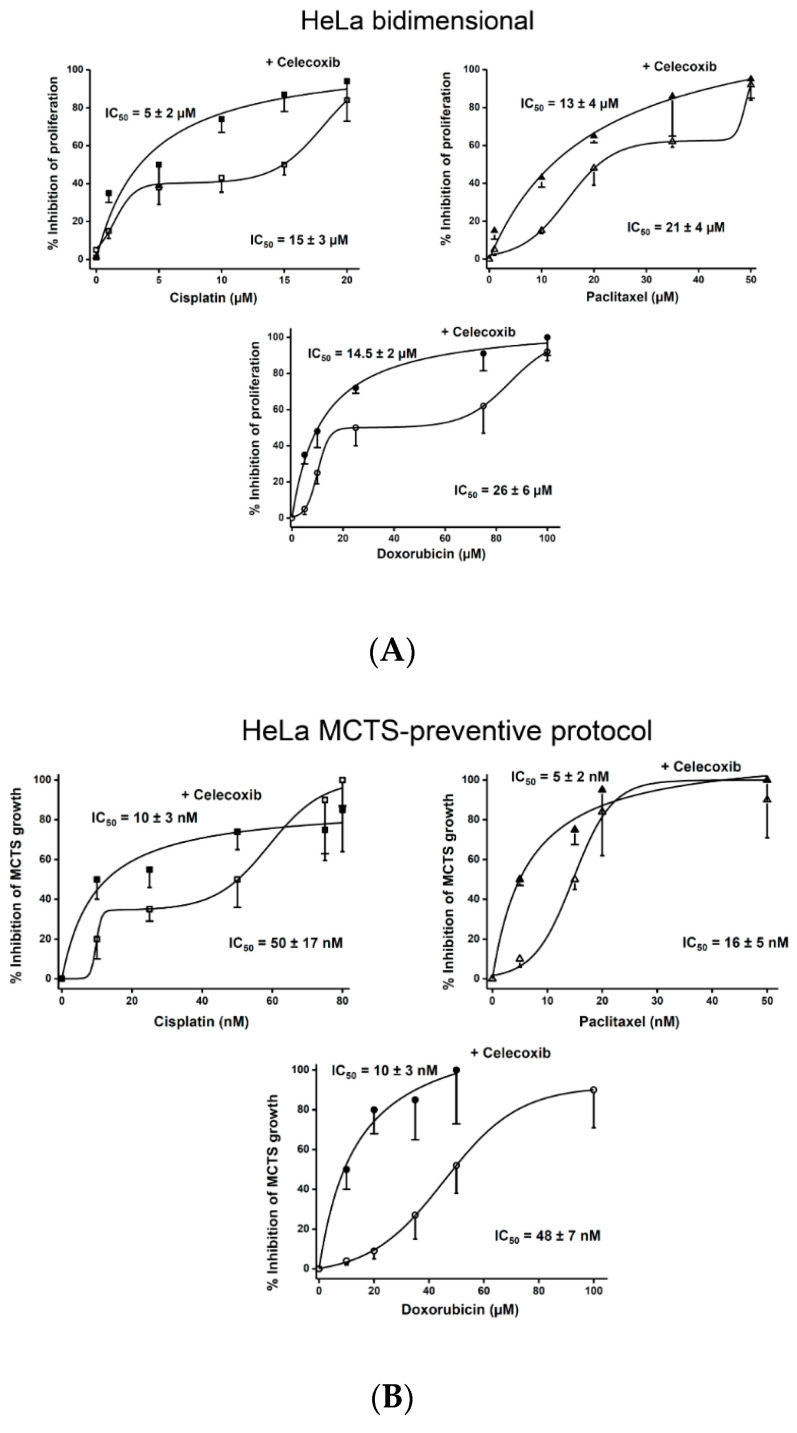

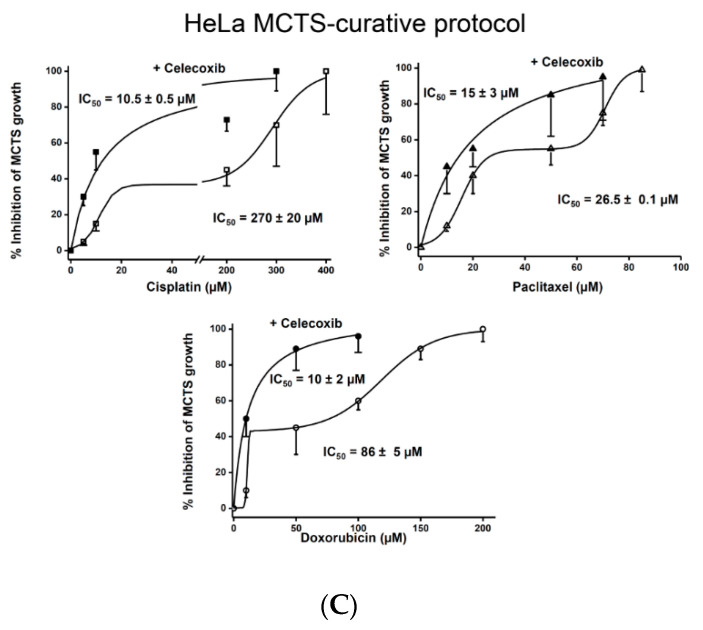
Celecoxib sensitizes HeLa bidimensional (**A**) cultures (*n* = 3) and MCTS under (**B**) preventive and (**C**) curative protocols (*n* = 30 MCTS) to cisplatin, paclitaxel, and doxorubicin. For bidimensional cultures, celecoxib was added at 5–10 µM. For MCTS, celecoxib was added at 0.4–1 nM or 2–6 µM in preventive or curative protocols, respectively.

Celecoxib (5–10 µM) and DMC (10–25 µM) synergistically increased the potency of canonical anti-cancer drugs on HeLa cell growth in both bidimensional and tridimensional cultures (Figure 2; Figure S2; Table 2). Likewise, a significant decrement (by 38–66%) of the cisplatin and doxorubicin IC_50_ values resulted with the presence of either NSAID in bidimensional cultures (Table 5). In HeLa MCTS, celecoxib in both the preventive and curative protocols also decreased the chemotherapy drug IC_50_ values, but it was by a greater extent (>70%) (Table 6).

DMC also enhanced the anti-cancer cytotoxicity of cisplatin and paclitaxel, lowering their IC_50_ values in both preventive (by 52–55%) and curative (by 45–73%) protocols (Table 6). CasII-gly also enhanced the toxicity of cisplatin, paclitaxel, and doxorubicin by decreasing their IC_50_ values in bidimensional (by 19–54%; Table 5) and tridimensional cultures (by 62–96%; Table 6). Similar to celecoxib, CasII-gly increased the cisplatin toxicity (by 84–96%), which was greater than that found with the other chemotherapy drugs.

### 2.4. Effect of Drug Combination on Growth of Non-Cancer Cells in Bidimensional Culture

As a mandatory, rigorous control, the effect of celecoxib and DMC as single agents or combined with cisplatin, paclitaxel, and doxorubicin was also assessed on the growth of non-cancer cells (3T3 mouse and HFF1 fibroblasts) (Table 1 and Table 5). To this end, the therapeutic index (TI) ratios were also determined. The combination of celecoxib or DMC with cisplatin, paclitaxel, or doxorubicin yielded TI values above 3 (Table 7) for both 3T3 or HFF1 fibroblasts, indicating that the drugs examined showed minimal toxicity toward these non-cancer cells but produced the desired effect on cancer cells [49].

### 2.5. Effects of Synergistic Drug Combinations on Mitochondrial Function and Invasiveness in Bidimensional HeLa Cells

In an initial attempt to understand why NSAIDs and cisplatin or paclitaxel produced a strong inhibition of HeLa cell proliferation, drugs added alone or in combination were assayed for their effects on cellular energy metabolism and cell invasiveness. Based on data from Table 2, the selected doses for each drug were 5 µM celecoxib, 2 µM cisplatin, and 15 µM paclitaxel.

**Figure 3:**
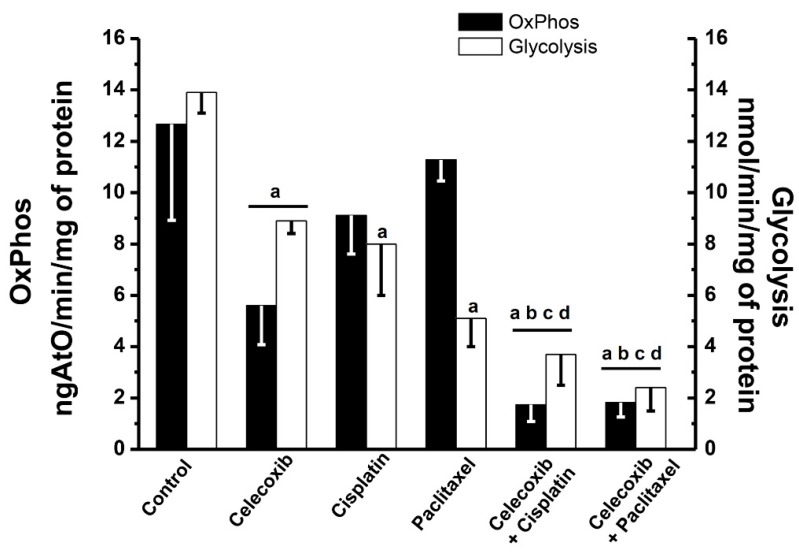
Effect of celecoxib (5 µM), paclitaxel (15 µM), and cisplatin (2 µM) added alone or in combination on OxPhos and glycolysis fluxes, after 24 h exposure in HeLa cells. The data show the mean ± S.D. of at least three different preparations. ^a^ *p* < 0.05 vs. control (no added drugs); ^b^ *p* < 0.05 vs. celecoxib; ^c^ *p* < 0.05 vs. cisplatin; ^d^ *p* < 0.05 vs. paclitaxel.

Paclitaxel or cisplatin alone did not significantly affect OxPhos flux whereas glycolysis was decreased by 40–60% (Figure 3). In turn, celecoxib alone inhibited both OxPhos and glycolysis by 36–50%. However, cisplatin or paclitaxel combined with celecoxib were able to decrease OxPhos flux by more than 80%; i.e., celecoxib induced a synergistic OxPhos inhibition with either cisplatin or paclitaxel. The effect of the celecoxib analogue DMC was also analyzed on OxPhos flux. DMC (15 µM) alone blocked OxPhos flux by 60%; whereas in combination with cisplatin or paclitaxel, it promoted a stronger OxPhos inhibition of 80–85% (Figure S3).

A potentiating celecoxib effect was not observed for glycolysis where combining NSAIDs with the canonical chemotherapy drugs rather yielded an algebraic sum of effects. Both celecoxib/cisplatin or celecoxib/paclitaxel combinations slightly decreased (26–40%) energy metabolism in 3T3 fibroblasts (Figure S4); however, these doses did not affect fibroblast proliferation (Table 1).

**Figure 4:**
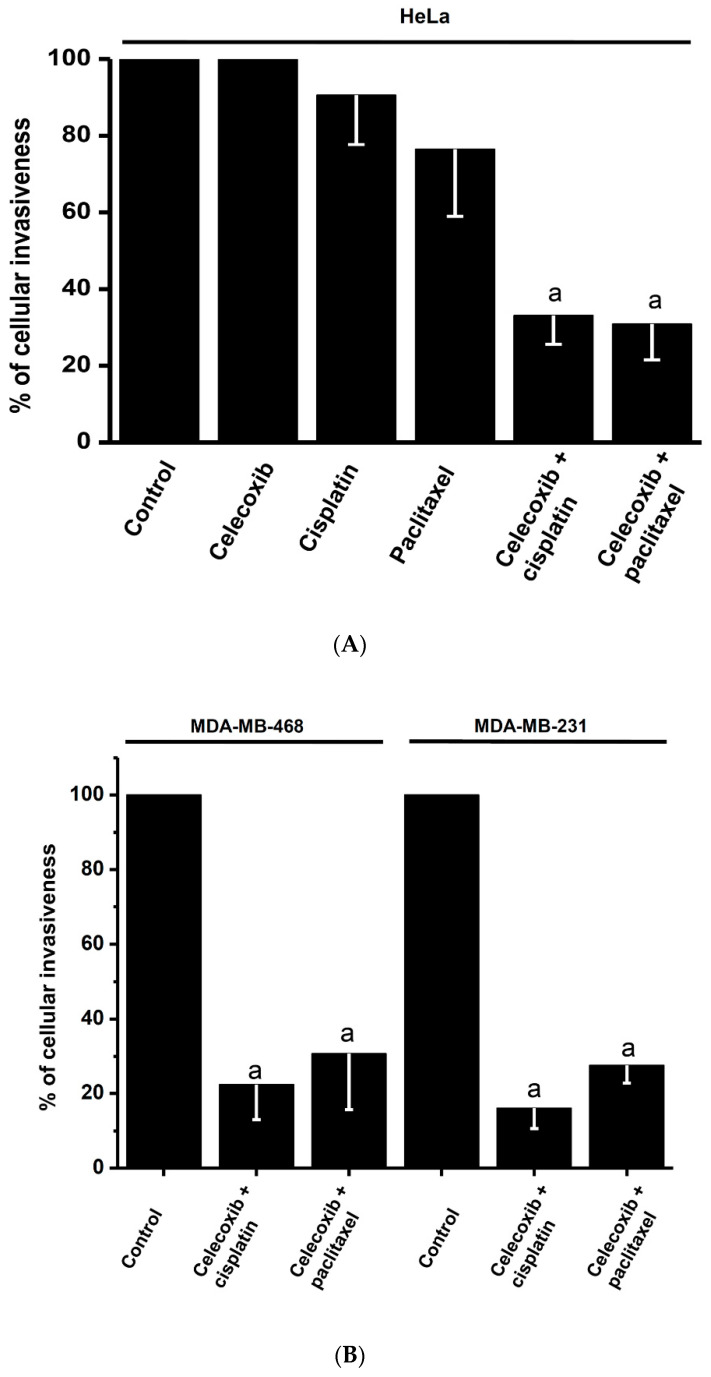
Effect of celecoxib (5 µM), paclitaxel (15 µM), and cisplatin (2 µM) and their combinations for 24 h on the invasiveness potential of HeLa (**A**) and metastatic breast cancer (**B**) cells. n = 3; ^a^ *p* ≤ 0.05 vs. control.

Cancer cell invasiveness is an energy-demanding cellular process [12]. HeLa cells maintain an invasiveness potential lower than that exhibited by the well-recognized metastatic cells MDA-MB-468 and MDA-MB-231 [50,51] (Figure S5). Celecoxib, paclitaxel, or cisplatin alone did not alter the HeLa cell invasiveness potential (Figure 4A). However, similarly to what was observed for OxPhos flux, combinations of celecoxib or DMC with the canonical chemotherapy drugs severely diminished the invasiveness potential of both HeLa (Figure 4A) and triple negative breast cancer (Figure 4B) cells by 60–80%.

### 2.6. Effects of Synergistic Drug Combinations on Cancer Growth, Mitochondrial Function, and Invasiveness in Bidimensional and Tridimensional SiHa and U373 Cells

In order to demonstrate whether a combination of celecoxib with canonical drugs also affects other cervix cancer lines, drugs were tested on the cellular growth and OxPhos flux of SiHa cells (Figure S6). The synergistic concentrations of celecoxib/paclitaxel or celecoxib/cisplatin used in bidimensional (Table 2) and tridimensional HeLa cell cultures, in both preventive and curative (Table 3) protocols, were very toxic for SiHa bidimensional and MCTS cell cultures (cellular viability decreased >70%), indicating that this cancer cell line is much less drug-resistant than HeLa cells [52,53]. Therefore, the IC_50_ proliferation values for each assayed drug were determined in SiHa bidimensional and MCTS cells. Thereafter, Bliss-type additivism analysis revealed that celecoxib (3 µM) combined with cisplatin (1 µM) showed a supra-additive effect (synergism was 50 ± 16%), impairing bidimensional SiHa growth and OxPhos flux (Figure S6) as occurs in HeLa cells. No synergism was found with celecoxib and paclitaxel. In SiHa MCTS cultures, celecoxib (0.1 nM–2 µM) combined with paclitaxel (10 nM–10 µM) or with cisplatin (10 nM–1 µM) showed supra-additive effects in both preventive (synergism was 44 ± 11%; n = 5 spheroids; data not shown) and curative (synergism was 70 ± 15%; Figure S6) protocols affecting SiHa MCTS growth.

Since the clinical stage of SiHa cells corresponds to advanced stage II, i.e., a low metastasis profile [54], drug combination analysis was also extended to another metastatic cancer cell (U373 glioblastoma) that represents the same clinical stage IV found in HeLa cells (Figure S7). The synergistic concentrations of celecoxib/paclitaxel or celecoxib/cisplatin used in HeLa bidimensional (Table 2) and MCTS (Table 3) cells also abolished the growth of U373 bidimensional (80 ± 9% and 83 ± 4% for celecoxib/cisplatin and celecoxib/paclitaxel, respectively) and MCTS in both preventive (>75% for both celecoxib combinations; data not shown) and curative (>90% for both celecoxib combinations, Figure S7) protocols. These synergistic drug combinations also blocked U373 OxPhos flux (80%) and invasiveness potential (77–85%), adding further support for combinatory drug therapy with NSAIDS to be used against metastatic cancer cells.

## 3. Discussion

Most of the chemotherapy drugs commonly used in clinical treatments show severe adverse side effects (for instance, cardiotoxicity, neuropathy, and nephrotoxicity), decreasing the patient’s quality of life [55,56,57]. Recently, cell-based assays and high-throughput drug screening technologies have allowed the faster identification of drugs targeting specific diseases [48]. In cancer, the synergistic effects from combinations of two or more drugs have been empirically discovered [58,59]. However, in several cases, these drug combinations are directed to the same target or to different targets but belong to the same or related pathways/processes [60,61], which has led to unsuccessful outcomes [62,63]. In other cases, clinicians use predictions reported in the literature that do not render synergy when administered to patients or alternatively, the drug combinations result in hematological toxicities [63,64,65].

### 3.1. NSAIDs as an Alternative to Decrease Proliferation of Cervical Cancer Cells

Several of the canonical anti-cancer drugs assayed in this study showed greater toxicity against HeLa cells than did the NSAIDs. Although the IC_50_ values were lower against 3T3 or HFF1 fibroblasts or MCF-10A breast epithelium cells, all of the chemotherapy drugs have shown significantly greater cardiotoxicity [42,66,67,68,69] than the NSAIDs which are presumably less harmful [70].

In the micromolar range, celecoxib or DMC used alone were able to block the cervical carcinoma HeLa cell proliferation in bidimensional cultures, similar to the levels that have also been reported for other metastatic cancer cells [12,71]. In the more realistic and physiological tridimensional model, celecoxib and DMC were also able to prevent MCTS formation (preventive protocol) and decrease the size of well-formed MCTS (curative protocol). Indeed, the NSAID doses required to decrease spheroid growth were one order of magnitude lower (in the nanomolar range) than those required to inhibit the growth of the bidimensional cultures. The difference in drug sensitivity between bi- and tridimensional models may be associated with the expression of the plasma membrane drug-expelling P-glycoprotein pump or with the cell architecture. It has been demonstrated that the P-glycoprotein expression is marginal in the early stages of MCTS growth (diameter = 100 µm) [72], which correlates with the low (nanomolar) drug concentration required to achieve cell death. In addition, small HeLa MCTS (350 µm diameter) have not yet reached a high cell density in their outer layers, facilitating drugs such as doxorubicin to be able to still diffuse from outer to inner (core) zones of small or “young” MCTS [73].

The celecoxib anti-proliferative effects might appear linked to its anti-COX-2 activity (COX-2 *Ki_celecoxib_* = 10 µM) [74] through the maintenance of a metastatic phenotype [14] and apoptosis activation [15,27]. Nevertheless, other essential pathways/functions such as OxPhos are also severely inhibited by low celecoxib doses [12,13]. Indeed, celecoxib affects OxPhos as well as the mitochondrial membrane potential (50–80% vs. non-treated cells) of HeLa cells [13]. These cervical cancer cells are highly dependent on OxPhos (>70%) for ATP supply under normoxia [75,76]. Accordingly, celecoxib is likely to inhibit HeLa cell proliferation in bi- and tridimensional cultures at doses where OxPhos [13] and glycolysis (Figure 3) become inhibited. OxPhos only provides approximately 15% of the total cellular ATP in mature MCTS of HeLa cells [75], because these spheroids develop large hypoxic inner areas. Therefore, OxPhos could be considered as a suitable and promising therapeutic target for the inhibition of cancer cell growth [77].

The celecoxib analogue, 2,5-dimethyl-celecoxib (DMC) lacks the COX-2 inhibitory function [27]: DMC at 100 µM blocks COX-2 activity by only 15% [30]. Nevertheless, DMC shows a potency similar to celecoxib for inhibiting cancer cell proliferation [24,27,29]. Similarly, HeLa cell proliferation in bi- and tridimensional cultures was also inhibited by DMC, as also reported for Burkitt’s lymphoma, myeloma, and non–small cell lung and gastric cancer cell growth [24,27,29]. Therefore, the DMC effects are most likely associated with COX-2 independent mechanisms. At micromolar (10–100 µM) doses, DMC induces apoptosis (down-regulating Bcl-2 proteins) and cell cycle arrest (up-regulating the cell cycle inhibitor p27) [30,46]. In addition, recent studies have demonstrated that DMC increases ROS production through OxPhos inhibition in different metastatic and low-metastatic cancer cell types [13].

### 3.2. Supra-Additive Effects of NSAIDs and Paclitaxel or Cisplatin Combinations on Cancer Cell Growth, Energy Metabolism, and Invasiveness

Several strategies have been developed to overcome drug toxicity [48,78]. One of them is related to the synergistic effect of one drug when combined with a second drug. Synergy may lead to using lower doses of both drugs, potentially avoiding unnecessary toxic side effects [48,79]. Several mathematical approaches have been proposed to describe the drug synergistic effect [80]. In the present study, two approaches, the Bliss-type additivism (BTA) and the resistance index (RI), were selected because both have been widely used in pharmacological tests with reproducible results [81]. Data were also analyzed by using the combination index (CI) approach [79]. By using BTA, RI, and CI, it became evident that NSAIDs induce potent synergistic effects on several chemotherapy drugs commonly used in cancer patient treatments and clinical trials [81]. 

The effects of combining celecoxib *plus* cisplatin, both used at doses similar to those reported here, have been documented for cervix (SiHa), lung (A431), vulva (SW962) carcinomas, and osteosarcoma (MG63). The main effects were an increased formation of DNA adducts, decreased cell proliferation, and greater apoptosis activation [18,82,83,84]. In addition, it has been documented that celecoxib (60 mg/kg) combined with auranofin (10 mg/kg), another anti-inflammatory drug used for rheumatoid arthritis, decreased by approximately 50% colon (DLD-1) cancer growth in in vivo mouse models [85]. Unfortunately, the latter studies did not explain whether the doses assayed for their drug combinations were lower than those of the individual IC_50_ values in order to reveal any potential synergistic effect for the drugs tested. Neither were the effects of these drug combinations analyzed in non-cancer cells. On the other hand, the combination of celecoxib/paclitaxel has been used in a preclinical, phase II study of lung cancer, with poor outcomes [86], but this could be related to the relatively short duration of the treatment.

The synergism of celecoxib with other chemotherapy drugs such as the multi-kinase inhibitor sorafenib [87] or with 5-Fluorouracil (5-FU) in patients bearing hepatocellular carcinoma [88] have been also documented. In the first example, a high synergism of 47–89% was obtained, although the drug combinations were not assayed on non-cancer cells. Celecoxib combined with sorafenib increased the extent of apoptosis, inactivated the MEK/ERKK signaling pathway, and hence, lowered cell viability of HepG2 and Huh7 hepatocarcinoma cells vs. sorafenib used alone [87]. In the second example, celecoxib combined with 5-FU increased the patient disease-free survival by 40% with a significant decrease (>80%) in vascular endothelial growth factor (VEGF) as a marker of malignancy. In combination, the assayed drug doses, were not lowered compared to the use of individual drug levels to allow for assessing any synergy effect [88].

In the present study, it is shown for the first time that celecoxib or DMC, when combined with cisplatin or paclitaxel, synergistically decreases proliferation of low metastatic (SiHa) and high metastatic (HeLa and U373) cells as well as their invasiveness ability in vitro, which was mediated at least partially by energy metabolism inhibition. Both glycolysis and OxPhos were drastically abolished by combinations of celecoxib with either paclitaxel or cisplatin. Particularly for OxPhos, celecoxib or DMC alone decreased the mitochondrial flux by 50–60%. These data correlated with previous results showing that celecoxib and DMC, and other drugs such as the vitamin E derivatives MitoVES and M-TEA, are potent respiratory chain inhibitors and/or uncouplers (i.e., H^+^ ionophores), promoting enhanced oxidative stress and with limited effects on non-cancer cells [12,13,89,90]. Nevertheless, other mechanisms at the transcriptional [91,92], translational, or signaling levels cannot be discarded.

In cancer cells, the inhibition of OxPhos by celecoxib or DMC should in turn impair all ATP-dependent processes. When these NSAIDs were combined with chemotherapy drugs, supra-additive effects were observed in which OxPhos inhibition was over 80%. A survey of the literature found no previous reports where cancer cell OxPhos has been synergistically inhibited with any combination of chemotherapy drugs. However, in colon DLD-1, HCT116, and HT-29 cancer cells, a combination of auranofin (1 µM) and celecoxib (10 µM) for 24 h induced a decrease of 30–65% in both OxPhos and glycolysis fluxes [85]. The use of other drugs targeting cancer mitochondria such as vitamin E analogues in combination with canonical chemotherapy requires further investigation. 

Synergism of the drug combinations was also found on HeLa and U373 cell invasiveness, which is a well-known ATP-consuming process [12]. This cell process was inhibited by more than 60% when these cells were treated with celecoxib and paclitaxel or cisplatin. In gastric cancer, paclitaxel (2.4 µM) slightly decreases its invasiveness potential by 25% after 48 h incubation [93]. There are no other studies in which paclitaxel or cisplatin combined with NSAIDs have been assayed on metastatic processes.

CasII-gly showed a strong synergistic effect on cancer cell growth with cisplatin, paclitaxel, and doxorubicin. However, CasII-gly is still under experimental development, and hence, it will take longer before approved use in humans. CasIII-ia, another member of the casiopeina´s family, has been recently tested in Phase I clinical trials against cancer [82]. However, the trial outcomes have not been published yet.

### 3.3. Infra-Additive Effect of NSAIDs and Carboplatin or Cyclophosphamide on Cancer Cell Growth

A strong infra-additive effect of celecoxib with carboplatin or cyclophosphamide was observed in both HeLa cell models. Indeed, such drug combinations have been previously clinically tested on ovarian and lung carcinoma, and pancreatic adenocarcinoma [85,86]. However, cancer progression was not arrested, and most of the cancer patients showed poor survival rates [79,94,95].

Perhaps due to such negative outcomes with celecoxib and carboplatin or cyclophosphamide proving relatively ineffective, further studies of ovarian/lung/pancreatic carcinomas were deterred. The present findings are consistent with poorer outcomes in that such drug combinations are expected to provide infra-additive responses. Further research is required to elucidate why celecoxib decreases the cytotoxicity of carboplatin/cyclophosphamide for cancer cells.

In this last regard, it is noted that CasII-gly showed a strong infra-additive effect with the vitamin E analogues (α-TOS and α-TEA), probably because these drugs interfere with each other by targeting the same sites/processes (i.e., mitochondrial function).

## 4. Materials and Methods

### 4.1. Drugs

Paclitaxel (microtubule depolymerization inhibitor); doxorubicin (topoisomerase II inhibitor); cisplatin, carboplatin, cyclophosphamide (alkylating agents); gemcitabine (DNA analogue); and dimethylcelecoxib and celecoxib were obtained from Sigma-Aldrich Chemical Co. (St Louis, MO, USA). Casiopeina II-gly (CasII-gly) was kindly donated by Dr. Lena Ruiz from Facultad de Química, UNAM, Mexico. All drugs assayed were dissolved in 70% ethanol/30% DMSO, except for cisplatin, doxorubicin, and CasII-gly, which were dissolved in distilled water. The maximal volume of vehicle added to the cells (10–50 µL) did not affect cellular viability.

### 4.2. Cancer Cell Lines

Human HeLa and SiHa cervix carcinoma, human MDA-MB-231 and MDA-MB-468 breast carcinomas, human U373 glioblastoma, human MCF10A breast epithelial cells, HFF1 foreskin fibroblasts and mouse 3T3 fibroblasts were obtained from the ATCC (American Type Culture Collection). HeLa, MDA-MB-231, MDA-MB-468, SiHa, U373 and MCF10A cell genotyping analyses, performed by the National Institute of Genomic Medicine (INMEGEN, Mexico City, Mexico), showed that these cell lines shared 80–100% (14 from 16 for HeLa; 14 from 14 for MDA-MB-231; 10 from 10 for MDA-MB-468; and 17 from 17 for MCF10A, 15 from 15 for U373, 10 from 11 for SiHa) alleles reported by the ATCC for their authentication. The viability of all cancer lines assayed, estimated by 0.5% trypan blue exclusion, was higher than 95%, and under drug exposure, it was higher than 85%.

### 4.3. Determination of Drug IC_50_ Values in Bidimensional Cultures

For cellular proliferation with and without drugs, the cell lines (20 × 10^3^ cells/well) were grown as bidimensional in 96-well plates containing Dulbecco’s Modified Eagle’s Medium (DMEM) (Sigma-Aldrich) for 24 h. Afterwards, celecoxib, DMC, CasII-gly, cisplatin, doxorubicin, paclitaxel, gemcitabine, cyclophosphamide (0.01, 0.1, 10 and 100 μM), or carboplatin (0.001, 0.01, 0.1, 1 and 2 mM) were added to the cell cultures and incubated for an additional 24 h. For SiHa and U373 cells, celecoxib, cisplatin, and paclitaxel were added to the cell cultures at final concentrations of 0.1, 1, 10, and 100 μM. The effect of these inhibitors on cell proliferation was determined by using the 3-(4,5-dimethyl-thiazol-2-yl)-2,5-diphenyltetrazolium bromide (MTT) assay (Sigma-Aldrich), which is a highly reliable and reproducible method compared with other cell proliferation tests, as described elsewhere [12]. The agents used for the MTT assay did not affect the viability of any of the assayed cancer cells (viability was >95%).

### 4.4. Multi-Cellular Tumor Spheroid (MCTS) Cultures

HeLa, SiHa, and U373 (1 × 10^5^ cells/mL) were seeded onto 2% (*w/v*) agarose-coated culture dishes in 5 mL DMEM. After 5 days, old medium was replaced with fresh DMEM, and spheroids were placed under slow orbital shaking (20–50 rpm) at 37 °C and 95% air/5% CO_2_. To discard incompletely formed spheroids, fresh DMEM was replaced every three days. The spheroid growth was determined at different culture days by measuring diameters using the calibrated reticule (1/10 mm) of an inverted phase contrast microscope (Zeiss, Thornwood, NY, USA) [75]. The growth of each MCTS was analyzed by fitting data to a logistic function for exponential growth using the Origin 8 software (Northampton MA, USA).

### 4.5. Determination of Drug IC_50_ Values in Tridimensional Spheroid Cultures

Two protocols for drug addition in the MCTS model were established: (a) “preventive protocol”—drug was added on the first day of culture (day 1); and (b) “curative protocol”—drug was added once the MCTS were formed, at which time the half of its maximal size was reached. These times were day 10 for HeLa MCTS of around 500 µm diameter and day 4 for SiHa and U373 MCTS of around 300 µm diameter. The IC_50_ values for MCTS growth were determined for both experimental protocols on day 20 (HeLa) or day 9 (SiHa or U373) of culture, at which MCTS maximal size was attained. Different concentration ranges were tested for either celecoxib (0.01, 0.1, 1, 10, 100 nM and 1, 10 µM), DMC (0.01, 1, 10, 100 nM and 1, 10, 50 µM), CasII-gly (0.01, 0.1, 1, 10, 100 nM and 1, 10, 100, 200 µM), or cisplatin, paclitaxel, doxorubicin, gemcitabine, cyclophosphamide, or carboplatin (0.01, 0.1, 1, 10, 100 nM and 1, 10, 100, 500 µM).

### 4.6. Analysis of Drug Toxicity by Assessing Bliss-Type Additivism, Resistance Index Ratio (RI), and Combination Index (CI) Value

To identify any synergy existing between the effects of celecoxib, DMC, or CasII-gly with those of different canonical anti-cancer drugs, three standard reference mathematical models were applied. For all models, each drug was added to the cells at sub-IC_50_ doses (as indicated in Table 2 and Table 4; and Table S1). The Bliss-type additivism model [48,96] predicts the additive response *C* for two single drugs with effects *A* and *B,* following the equation*: C = A + B − (A × B),* where each effect is expressed as a fractional inhibition. The synergistic effect of the combined A and B drugs surpassing the monotherapy effect of each individual drug is determined from the difference between the experimentally determined effect produced by the drug combination and the C values derived from the Bliss-type additivism equation. The Bliss Independence model is considered as one of the most popular models to assess the combined effects of drugs [97]. In this last model, three outcomes are identified: (a) *the infra-additive effect,* in which both drugs exerted a null response on cellular growth (or even stimulated it); (b) *the additive effect,* in which the combined effect produced by two or more drugs is the algebraic sum of their separate effects; and *(c) the supra-additive (also called potentiation or synergism) effect,* in which the combined effect produced by two or more drugs is higher to the sum of their separate effects [48,82].

The combination effect of NSAIDs and chemotherapy drugs on proliferation was also analyzed by determining the resistance index ratio (RI) and the combination index (CI) value. RI was calculated as the ratio of expected cell survival (Sexp, defined as the product of the survival observed with drug A alone and the survival observed with drug B alone) to the observed cell survival (Sobs) for the combination of A and B (*RI = Sexp/Sobs)*. Accordingly, an RI value higher than 2 indicates a clear drug synergistic effect [98]. CI was calculated by using the response additivity approach [79] and applying the mathematical formula *CI = (DA + DB)/DAB*, where DA and DB represent the effects of two individual drugs and DAB represents the effect of the drug combination. Accordingly, CI values lower than 1 indicate a supra-additive (synergistic) effect [79].

### 4.7. Therapeutic Index Ratio (TI Ratio)

TI values were determined by dividing the IC_50_ value attained for non-cancer cells into the IC_50_ attained for cancer cells. A TI >3 indicates that the exposure to the drug results in low or null toxicity for normal cells and high toxicity for cancer cells [49].

### 4.8. Determination of Glycolytic and OxPhos Fluxes in Bidimensional Cancer and Non-Cancer Cells Exposed to Drug Combinations

For glycolytic flux, HeLa cells or 3T3 fibroblasts (1–3 mg protein/mL), cultured for 24 h with the indicated drugs, were harvested and further incubated in Krebs-Ringer (KR) buffer at 37 °C under smooth orbital shaking as previously reported [99]. Glycolysis was started by adding 5 mM glucose (Sigma-Aldrich). Cellular samples were collected after 0 and 10 min of incubation, swiftly mixed with 30% (*w/v*) of cold perchloric acid, and centrifuged at 3500 rpm for 5 min. Supernatants were neutralized with 1N KOH/100 mM Tris. Cells were also incubated with 2-deoxyglucose (2-DG, 20 mM) (Sigma-Aldrich) to prevent lactate production by glycogen degradation. Lactate production was determined by the lactate dehydrogenase (Roche, Mannheim, Germany) coupled assay registering the NADH formation at 340 nm [100].

For total oxygen consumption and OxPhos fluxes, HeLa, SiHa, or U373 cells or 3T3 fibroblasts (1 mg protein/mL), cultured for 24 h with the indicated drugs, were harvested and further incubated at 37 °C in air-saturated KR buffer and under constant agitation. To discard non-mitochondrial oxygen consumption [101], the cells were incubated with 5 μM oligomycin (Sigma-Aldrich), a specific and permeable inhibitor of the mitochondrial ATP synthase. The OxPhos flux (i.e., the rate of oligomycin-sensitive oxygen consumption) was determined by using a Clark type electrode, as described elsewhere [101], and a high-resolution respirometer (Oroboros Instruments, Innsbruck, Austria).

The contribution of glycolysis and OxPhos to the cellular ATP supply was calculated, respectively, from the rate of 2-DG-sensitive lactate production, assuming a stoichiometry of 1 mol of ATP produced per 1 mol of lactate generated, and from the oligomycin-sensitive respiration rate multiplied by the ATP/O ratio of 2.5 for cancer cells [102]. Trypan blue assay revealed >95% cellular viability in all cancer cells assayed before drug treatment. After drug exposure, viability was >85% for HeLa, SiHa, and U373 cells.

### 4.9. Cell Invasiveness Assays

HeLa, U373, MDA-MB-231, or MDA-MB-468 cells (5 × 10^4^ cells/mL) were suspended in 0.1 mL serum-free DMEM and placed on the upper compartment of Boyden chambers (Trevigen Inc., Helgerman CT, Gaithersburg, MD, USA) in the presence of individual drugs (5 µM celecoxib; 10 µM cisplatin; or 15 µM paclitaxel) or their combinations as indicated. The lower compartment was filled with serum-free DMEM. After 24 h at 37 °C, the invasive cells found in the lower compartment were loaded with 60 nM calcein AM (acetomethylester) for 60 min. Calcein fluorescence was detected at λ_exc_ = 485 nm and λ_em_ = 520 nm by using a microplate reader (Varioskan Lux, Thermo Fisher Scientific; Waltham, MA, USA). The metastatic breast cancer MDA-MB-231 and MDA-MB-468 cells (5 × 10^4^/mL) were used as invasive cell controls [103]. Trypan blue assay revealed >95% cellular viability in all cancer cells assayed before drug treatment. After drug exposure, viability was >85% for HeLa, SiHa, and U373 cells.

### 4.10. Statistics

Data were expressed as mean ± standard deviation (S.D.). For statistical comparison between two independent experimental groups (Student´s *t*-test) and among more than two experimental groups, appropriated statistical tests were assayed (ANOVA and post hoc Scheffe tests) [104,105], considering *p* < 0.01 and *p* < 0.05 as criteria of significance.

## 5. Conclusions

The data of the present study indicate that to effectively inhibit HeLa, SiHa, and U373 cancer cell growth with limiting side effects on non-cancer cells, an approach that simultaneously targets essential and highly active cancer cell functions such as OxPhos (with celecoxib/DMC) and DNA/microtubule stability (with cisplatin/paclitaxel) or topoisomerase II activity (with doxorubicin) may not only be successful under controlled laboratory conditions, but may also help in the clinical setting. Indeed, the present results provide support for improving clinical strategies specifically targeting cervical carcinomas. Thus, the aim will be to re-purpose drugs such as NSAIDs as adjuvant therapy for use in combination with canonical chemotherapy drugs. Hence, the urgent need for repositioning celecoxib for the specific treatment of cervix cancer [106] should become a public health priority in the near future.

## Figures and Tables

**Table 1:** IC_50_ (μM) values for non-steroidal anti-inflammatory drug (NSAIDs), canonical and non-canonical chemotherapy drugs alone on cancer and non-cancer cell growth.

Bidimensional Cell Cultures					Tridimensional HeLa MCTS	
**Drugs**	**Cancer**	**Non-cancer**			**Preventive (nM)**	**Curative (μM)**
	HeLa	3T3	MCF-10A	HFF1		
	***NSAIDs***					
Celecoxib	55 ± 9 ^a,b,c^	119 ± 7	>100	96 ± 13	1 ± 0.3	7.5 ± 2
Dimethyl Celecoxib (DMC)	48 ± 2 ^a,b^	69 ± 8.5	>100	44 ± 11	10 ± 2	32 ± 10
	***Non-canonical Drug***					
CasII-gly	1.5 ± 0.9 ^a,b^	9 ± 2	17 ± 3	N.D.	30 ± 7.5	106 ± 2
	***Canonical Anti-cancer Drugs***					
Cisplatin	15 ± 3 ^a,b,c^	36 ± 3	82 ± 4	59 ± 26	50 ± 17	270 ± 20
Paclitaxel	21 ± 4 ^a,b^	80 ± 12	100 ± 18	68 ± 30	16 ± 5	26.5 ± 0.1
Doxorubicin	26 ± 6 ^b,c^	51 ± 18	82 ± 25	65 ± 19	48 ± 7	86 ± 5
Gemcitabine	30 ± 5 ^a,b^	>2 mM	>1 mM	N.D.	65 ± 17	128 ± 20
Cyclophosphamide	16 ± 3 ^a,b^	>1 mM	102 ± 35	N.D.	136 ± 87	315 ± 30
Carboplatin	1 ± 0.4 ^a^ mM	>5 mM	>1 mM	N.D.	165 ± 28	287 ± 43

The IC_50_ values (μM, unless otherwise indicated) for the bidimensional cultures were determined after 24 h of drug exposure. For MCTS, the IC_50_ values were determined at day 20 of growth. The data shown represent the mean ± S.D. of at least three different independent bidimensional (*n* = 3) or tridimensional (*n* = 3) cultures. From each tridimensional culture, 10 spheroids were analyzed (total = 30 spheroids). N.D., not determined. ^a^ *p* < 0.05 vs. 3T3. ^b^ *p*< 0.05 vs. MCF-10A, ^c^ *p* < 0.05 vs. HFF1.

**Table 2:** Synergistic effects of NSAIDs with canonical anti-cancer drugs at sub-IC_50_ concentrations in bidimensional HeLa cell cultures—Bliss-type additivism.

*Drug 1.*	*Assayed Doses* (µM)	*Drug 2*	*Assayed Doses* (µM)	*C Values (BTA %) (Range)*	*Experimental Values (%) (Range)*	*Synergism (%)* *(Range)*
Celecoxib	5–10	Cisplatin	2–5	15.5 ± 4.5 (10–19)	81 ± 14 (65–91)	**66 ± 10 (55–74]**
	5–10	Paclitaxel	11–15	15 ± 3 (11–17)	84 ± 5 (81–90)	**69 ± 8 (64–79]**
	5–10	Doxorubicin	10–20	14 ± 3 (11–17)	71 ± 11 (58–79.5)	**57 ± 13 (42–68)**
	5–10	Gemcitabine	14–17	9 ± 3 (5–11)	55.5 ± 6 (48–59)	47 ± 8 (37–54)
	5–10	Cyclophosphamide	10–20	9 ± 4 (4–13)	−(69 ± 28) (−(44–99))	−(78 ± 24) (−(57–103))
	5–10	Carboplatin	5–15	7 ± 3 (4–10)	−(20 ± 8) (−(13.5–29))	−(27 ± 11) (−(18–39))
DMC	10–15	Cisplatin	2–5	15.5 ± 4.5 (10–19)	69 ± 4 (65–74)	**54 ± 5 (48–58)**
	20–25	Paclitaxel	20–21	15 ± 3 (11–17)	52.5 ± 3 (49–54)	38 ± 0.8 (37–38)
	20–25	Doxorubicin	10–20	14 ± 3 (11–16)	52 ± 7 (45.5–60)	38 ± 10 (30–48)
	10–25	Gemcitabine	10–17	16 ± 15 (5–34)	49 ± 0.6 [48–50)	33 ± 16 (15–45)
	10–25	Cyclophosphamide	10–20	9 ± 4 (4–12)	−(17 ± 7) (−(10–25))	−(26 ± 10) (−(20–37))
	10–25	Carboplatin	5–15	7 ± 3 (4–10)	−(37 ± 5.5) (−(33–43))	−(44 ± 3.5) (−(40–47))
CasII-gly	0.5–1	Cisplatin	5–10	15.5 ± 4.5 (10–19)	54.5 ± 3 (51.5–56.5)	39 ± 7 (32–46)
	0.5–1	Paclitaxel	10–20	15 ± 3 (11–17)	56.5 ± 4 (53–61.5)	42 ± 8 (36–50)
	0.5–1	Doxorubicin	10–20	14 ± 3 (11–16)	69 ± 6 (63–75)	**55 ± 8 (47.5–64)**
	0.3–1	Gemcitabine	5–10	16 ± 15 (5–34)	40 ± 10 (33–51.5)	24 ± 7 (18–31)
	0.3–1	Cyclophosphamide	25–35	9 ± 4 (4–12)	58 ± 10 (48–67)	49 ± 10 (38–55)
	0.3–1	Carboplatin	150	7 ± 3 (4–10)	58 ± 6 (51–64)	**51 ± 9 (41–59)**

The Bliss-type additivism (BTA) was calculated as outlined in the Section 4. Drug synergism, i.e., supra-additive effect when positive values are produced or infra-additive effect when negative values are produced, was calculated from the difference between the experimental values and the BTA *C* values. The bold range indicates stronger drug synergy was achieved when combining NSAIDs or CasII-gly with canonical drugs.

**Table 3:** Synergistic effects of NSAIDs with canonical anti-cancer drugs at sub-IC_50_ concentrations in bidimensional HeLa cell cultures—Resistance index.

*HeLa Bidimensional Cultures*					*3T3 Bidimensional Cultures*
*Drug 1*	*Assayed Doses* (µM)	*Drug 2*	*Assayed Doses* (µM)	*RI Value (Range)*	*RI Value (Range)*
Celecoxib	5–7	Cisplatin	1–5	**13 ± 11 (4.4–28)**	1.4 ± 0.7 (0.5–2.1)
	5–7	Paclitaxel	11–15	4 ± 2 (3.6–6)	0.8 ± 0.2 (0.6–0.9)
	5–10	Doxorubicin	15–20	7 ± 6 (3.7–16.5)	1.3 ± 0.4 (0.9–1.7)
DMC	10–15	Cisplatin	4–5	6 ± 4 (3.1–12.6)	1.2 ± 0.4 (0.7–1.4)
	20–25	Paclitaxel	20–21	7 ± 3.5 (4–11)	0.5 ± 0.2 (0.3–0.6)
	25	Doxorubicin	17	3 ± 0.4 (2.9–3.5)	0.4 ± 0.1 (0.3–0.5)
CasII-gly	0.5–1	Cisplatin	5–10	**18 ± 23 (5–61)**	0.4 ± 0.1 (0.3–0.5)
	0.5–1	Paclitaxel	10–20	**10 ± 11 (3.2–27)**	0.5 ± 0.3 (0.3–0.8)
	0.5–1	Doxorubicin	13	5 ± 4 (6.6–11)	0.5 ± 0.1 (0.3–0.65)
*MCTS Preventive Protocol*					
*Drug 1*	*Assayed Doses* (nM)	*Drug 2*	*Assayed Doses* (nM)	*RI Value (Range)*	
Celecoxib	0.4–0.7	Cisplatin	10–30	3 ± 1 (2.2–5.5)	
	0.1–0.9	Paclitaxel	10–13	3 ± 0.8 (2.3–4.7)	
	0.1–0.5	Doxorubicin	40	4.5 ± 2 (2.9–7.3)	
DMC	1–7	Cisplatin	30	6 ± 2 (3.6–8.3)	
	5	Paclitaxel	10–13	6 ± 3 (3.1–8.7)	
	1	Doxorubicin	30–40	3 ± 0.4 (2.3–3)	
CasII–gly	10–15	Cisplatin	30	4 ± 1 (2–5)	
	2–3	Paclitaxel	13	4 ± 3 (2–8)	
	20–25	Doxorubicin	25	4 ± 2 (2–7)	
*MCTS Curative Protocol*					
*Drug*	*Assayed Doses* (µM)	*Chemotherapy drugs*	*assayed doses* (µM)	*RI Value (Range)*	
Celecoxib	2–5	Cisplatin	3–5	4 ± 2 (2–7.4)	
	2	Paclitaxel	15–20	**7 ± 5 (2–15)**	
	4	Doxorubicin	30–50	5 ± 3 (2–8)	
DMC	10–25	Cisplatin	2–5	4 ± 2 (2–6.9)	
	20	Paclitaxel	20–25	4 ± 2 (4–6)	
	35	Doxorubicin	50	3 ± 1 (1.8–4)	
CasII-gly	11–12	Cisplatin	30	6 ± 4 (3–11)	
	20	Paclitaxel	15	1 ± 0.1 (0.9–1.3)	
	30	Doxorubicin	10	3 ± 0.8 (2–4)	

The data shown represent the mean ± S.D. of at least three different independent bidimensional (*n* = 3) or tridimensional (*n* = 3) cultures. From each tridimensional culture, 10 spheroids were analyzed (total = 30 spheroids). The bold range indicates stronger drug synergy was achieved when combining celecoxib or CasII-gly with cisplatin or paclitaxel.

**Table 4:** Synergistic effects of NSAIDs with canonical anti-cancer drugs on HeLa MCTS growth.

Preventive Protocol						
***Drug 1***	***Assayed Doses* (nM)**	***Drug 2***	***Assayed Doses* (nM)**	***C Values (BTA %) (Range)***	***Experimental Values (%)*** ***(Range)***	***Synergism (%)*** ***(Range)***
Celecoxib	0.4–0.7	Cisplatin	10–43	30.5 ± 4 (26–33)	93 ± 3 (91–95.5)	**62 ± 6 (59–69)**
	0.1–1	Paclitaxel	10–13	9 ± 1 (8–10)	83 ± 0.7 (82.5–84)	**74.5 ± 1 (74–76)**
	0.1–0.5	Doxorubicin	20–40	31 ± 3 (27.5–33)	83 ± 5 (77–86.5)	**52 ± 8 (43–58)**
	0.1–1	Gemcitabine	30–50	34 ± 6 (27–40)	−(23 ± 7) (−(16.5–30))	−(58 ± 12) (−(44–66))
	0.1–1	Cyclophosphamide	10–100	20 ± 21 (4–44)	−(37 ± 25.5) (−(11–62))	−(57 ± 8) (−(51–66))
	0.1–1	Carboplatin	100	19 ± 3 (17–22.5)	33 ± 3 (29–34.5)	14 ± 2.5 (12–16.5)
DMC	1–7	Cisplatin	10–43	33 ± 5 (29–38)	81 ± 3 (79–85)	48 ± 8 (41–56)
	5–6	Paclitaxel	10–13	13 ± 7 (5–17.5)	31 ± 5 (26.5–36)	18 ± 5 (12–22)
	1–3	Doxorubicin	25–40	19 ± 10 (11–30.5)	62.5 ± 6 (59–69)	43 ± 5 (39–48)
	1–10	Gemcitabine	30–50	33 ± 2 (31–34)	58 ± 5 (53–63)	25 ± 5 (20–28)
	1–10	Cyclophosphamide	10–100	35 ± 5 (31–41)	20 ± 6 (−(13–24)	−(15 *± 7)* (−(7.5–20))
	1–10	Carboplatin	10–100	40.5 ± 2 (38–42)	19 ± 0.3 (−(18–19))	−(21 ± 1.5) (−(20–23))
CasII-gly	10–17	Cisplatin	15–30	14 ± 4 (11–18.5)	95 ± 0.5 (94–96)	**81 ± 4.5 (76–85)**
	14–25	Paclitaxel	10–13	25 ± 2 (24–28)	67 ± 5 (63–72)	41.5 ± 2 (39–44)
	10–25	Doxorubicin	20–40	31 ± 9 (22–39)	62 ± 9 (52–69)	31 ± 2 (30–34)
	10–25	Gemcitabine	10–40	25 ± 0.6 (25–26)	54 ± 4 (51–59)	29 ± 4 (26–33)
	5–25	Cyclophosphamide	28–110	25 ± 2 (23–28)	10 ± 5 (5–13.5)	−(15 ± 3.5) (−(11.5–18.5))
	1–30	Carboplatin	50–90	33 ± 4 (28.5–37)	68 ± 7.5 (62–76)	35 ± 5 (29–39)
**Curative Protocol**						
*Drug 1*	*Assayed Doses* (µM)	*Drug 2*	*Assayed Doses* (µM)	*C Value (BTA%) (Range)*	*Experimental Values (%)* *(Range)*	*Synergism (%)* *(Range)*
Celecoxib	2–5	Cisplatin	1–5	15.5 ± 4.5 (10–19)	83 ± 12 (68.5–91)	**67 ± 8 (58–74)**
	2–6	Paclitaxel	10–25	23 ± 4 (20–28)	40 ± 8 (32–46.5)	17 ± 7 (10–23)
	2–4	Doxorubicin	30–50	23 ± 4 (19–27)	59 ± 3 (56–63)	37 ± 5 (32–41)
	1–5	Gemcitabine	20–50	29 ± 4 (24–32)	24 ± 7 (16.5–31)	−(4 ± 3) (−(1–8))
	4–7	Cyclophosphamide	75–115	27 ± 1.5 (26–29)	23 ± 3.5 (19–25)	−(4 ± 2) (−(2–7))
	1–7	Carboplatin	60–190	45 ± 5 (40–48)	25 ± 13 (16–40)	−(20 ± 11) (−(7–29))
DMC	10–25	Cisplatin	1–5	24 ± 4 (22–28)	71 ± 5 (66–75)	47 ± 4 (44–51)
	20–30	Paclitaxel	10–25	33 ± 5 (29–38.5)	52.5 ± 3 (49–54)	20 ± 8 (10–25)
	31–35	Doxorubicin	30–50	29 ± 9.5 (19–38)	52 ± 7 (45.5–59)	23 ± 16 (7–40)
	31–35	Gemcitabine	20–50	30 ± 2 (28–32)	20 ± 5) (16–25)	−(10 ± 3) (−(7–13))
	29–30	Cyclophosphamide	75–115	39 ± 6 (32–43)	14.5 ± 5) (8–18)	−(24 ± 10) (−(14.5–35))
	22–25	Carboplatin	60–190	24.5 ± 4 (20–27)	36 ± 0.9 (35–36.5)	11 ± 3 (9–14)
CasII-gly	10–100	Cisplatin	15–30	23 ± 3 (20–25)	97 ± 0.5 (97–97.5)	**74 ± 2 (72–76.5)**
	20–60	Paclitaxel	10–25	15 ± 3 (11–17)	64 ± 5 (61–69)	**50 ± 8 (44–58)**
	20–80	Doxorubicin	10	15 ± 4 (10–18)	46 ± 2 (44.5–48)	31 ± 6 (28–38)
	50–90	Gemcitabine	20–40	24 ± 16 (5–34)	65 ± 6 (58–70)	40.5 ± 11 (32–53)
	50–120	Cyclophosphamide	10–80	28 ± 2 (26–30)	19 ± 3 (17–23)	−(9 ± 5) (−(3–13))
	20–120	Carboplatin	50–75	29 ± 2 (28–31)	58 ± 6 (51–64)	29 ± 6 (23.5–36)

The data shown represent the mean ± S.D. of at least three different independent tridimensional cultures. From each culture, 10 spheroids were analyzed (total = 30 spheroids). The bold range indicates stronger drug synergy was achieved when combining celecoxib or CasII-gly with canonical drugs.

**Table 5:** Effects of NSAIDs or CasII-gly on the IC_50_ values of canonical anti-cancer drugs for growth of HeLa cells and 3T3 and HFF1 fibroblasts in bidimensional cultures.

Chemotherapy Drug	HeLa	3T3	HFF1
	+ Celecoxib (5–10 µM)		
Cisplatin	5 ± 2 ^a^	36 ± 4	75 ± 12
Paclitaxel	13 ± 4	52 ± 10	73 ± 18
Doxorubicin	14.5 ± 2 ^a^	54 ± 3	71 ± 7.3
	+ DMC (15–25 µM)		
Cisplatin	5 ± 1 ^a^	28.5 ± 7	74.5 ± 9.5
Paclitaxel	13 ± 4	58.5 ± 17	66.5 ± 8.5
Doxorubicin	11 ± 4 ^a^	41 ± 5	62.5 ± 1.5
	+ Cas-IIgly (0.5–1 µM)		
Cisplatin	9 ± 3	7.5 ± 6	N.D
Paclitaxel	17 ± 5	50 ± 20	N.D
Doxorubicin	12 ± 4 ^a^	38 ± 3	N.D

IC_50_ values were calculated after 24 h incubation and represent the mean ± S.D. of at least three different preparations. N.D., not determined. ^a^ *p* < 0.05 vs. anti-cancer drug monotherapy.

**Table 6:** Effects of NSAIDs or CasII-gly on IC_50_ values of canonical drugs for HeLa MCTS growth.

	**Preventive Protocol**			
**Canonical Drug**	**IC_50_ (nM)**	**+ Celecoxib** **(0.4–1 nM)**	**+ DMC** **(1–10 nM)**	**+ CasII-Gly** **(11–30 nM)**
Cisplatin	50 ± 17	10 ± 3 ^a^	24 ± 6 ^b^	8 ± 3 ^a^
Paclitaxel	16 ± 5	5 ± 2 ^b^	7.2 ± 3 ^b^	4.5 ± 1.5 ^a^
Doxorubicin	48 ± 7	10 ± 3 ^a^	31.2 ± 9	16.5 ± 4 ^b^
	**Curative Protocol**			
**Canonical Drug**	**IC_50_ (µM)**	**+ Celecoxib** **(2–6 µM)**	**+ DMC** **(10–35 µM)**	**+ CasII-gly** **(11–30 µM)**
Cisplatin	270 ± 20	10.5 ± 0.5 ^a^	148.5 ± 30 ^b^	11.3 ± 4 ^a^
Paclitaxel	26.5 ± 0.1	15 ± 3 ^b^	7.1 ± 2 ^a^	10 ± 2 ^b^
Doxorubicin	86 ± 5	10 ± 2 ^a^	77.4 ± 20	30 ± 9 ^a^

The IC_50_ values shown the mean ± S.D. of at least 3 different preparations. The NSAIDs and CasII-gly concentration ranges used are indicated. ^a^ *p* < 0.01 vs. anticancer drug monotherapy; ^b^ *p* < 0.05 vs. monotherapy.

**Table 7:** Therapeutic Index Ratio (TI ratio) for drug combinations in bidimensional non-malignant *versus* cancer cell cultures.

NSAIDs	NSAIDs Concentrations (µM)	Chemotherapy Drugs	TI Ratio	
			3T3/HeLa	HFF1/HeLa
Celecoxib	5	Cisplatin	36/5 = 7.2	75/5 = 15
	5	Paclitaxel	52/13 = 4.0	73/13 = 5.6
	10	Doxorubicin	54/14.5 = 3.7	71/14.5 = 4.9
DMC	15	Cisplatin	28.5/5 = 5.7	74.5/5 = 14.9
	20	Paclitaxel	58.5/13 = 4.5	66.5/13 = 5.1
	25	Doxorubicin	41/11 = 3.6	62.5/11 = 5.7

TI ratios were calculated from the IC_50_ values of each chemotherapy drug shown in Table 5, which were obtained in the presence of a constant concentration of the indicated NSAID. TI corresponds to the quotient of the IC_50_ values for mouse 3T3 fibroblasts or HFF1 fibroblasts divided by the IC_50_ values for HeLa cells; *n* = 3 different preparation.

## Supplementary Materials

The following are available online at https://www.mdpi.com/1424-8247/13/12/463/s1. Figure S1: Representative drug matrixes showing the effect of cisplatin plus celecoxib on HeLa cell cultures. Figure S2: Logarithmic dose-response curve shows the effect of cisplatin, paclitaxel and doxorubicin on HeLa bidimensional cultures (*n* = 3) and MCTS under preventive and curative protocols (*n* = 30 MCTS), with the presence of celecoxib. Figure S3: Effect of DMC (15 µM), paclitaxel (20 µM) and cisplatin (5 µM) alone or in combination, on OxPhos fluxes, after 24 h exposure in HeLa cells. Figure S4: Effect of celecoxib (5 µM), paclitaxel (15 µM) and cisplatin (2 µM) added in combination on OxPhos and glycolysis fluxes, after 24 h exposure in mouse 3T3 fibroblast. Figure S5: Invasiveness potential of different metastatic cancer cell lines. Figure S6. (A) Effect of celecoxib (3 µM) and cisplatin (1 µM) added in combination on cell proliferation and OxPhos flux, after 24 h exposure in SiHa bidimensional cultures. Control cell proliferation (100%) corresponded to 34 × 103 cells after 48 h culture. OxPhos flux control (100%) corresponded to 10 ± 1.5 ng At O/min/mg of protein. The data shown represent the mean ± S.D. of at least three different preparations for bidimensional culture. ^a^ *p* < 0.05 vs. control (no added drugs). Figure S7. Effect of celecoxib (5 µM), cisplatin (2 µM) and paclitaxel (15 µM) added in combination on (A) cell proliferation, OxPhos flux and (B) invasiveness after 24 h exposure in U373 bidimensional cultures. Control cell proliferation (100%) corresponded to 48 × 103 cells. OxPhos flux control (100%) corresponded to 13 ± 5 ng At O/min/mg of protein. The data show the mean ± S.D. of at least three different preparations for bidimensional culture. ^a^ *p* < 0.05 vs. control (no added drugs). (C) Effect of celecoxib (4-5 µM), cisplatin (1 µM) and paclitaxel (25 µM) added in combination on U373 MCTS growth using curative protocol (*n* = 5 spheroids). Table S1. Infra-additive effects of celecoxib, DMC or CasII-gly with vitamin E analogues in HeLa cell two-dimensional cultures and HeLa MCTS cultures. Table S2. Synergistic effects of NSAIDs with canonical anti-cancer drugs at sub-IC50 concentrations in bidimensional HeLa cell cultures as shown by using the Combination Index (CI) value.
