# Supplementary material for: Non-Steroidal Anti-Inflammatory Drugs Increase Cisplatin, Paclitaxel, and Doxorubicin Efficacy against Human Cervix Cancer Cells
Source: Pharmaceuticals (Basel). 2020 Dec 15;13(12):463. doi: 10.3390/ph13120463 (PMC7765098; doi:10.3390/ph13120463)
Supplement: Supplementary file 1 [file pharmaceuticals-13-00463-s001.pdf]

Nonsteroidal anti-inflammatory drugs increase cisplatin, paclitaxel and doxorubicin efficacy against human cervix cancer cells

Diana Xochiquetzal Robledo-Cadena[1], Víctor Dávila-Borja[2], Silvia Cecilia Pacheco-Velázquez[1], Stephen J. Ralph[3], Betsy Alejandra Blanco-Carpintero[1], Javier Alejandro Belmont-Díaz[1], Juan Carlos Gallardo- Pérez[1], Rafael Moreno-Sánchez[1] and Sara Rodríguez-Enríquez[1,*]

Table S1

Infra-additive effects of celecoxib, DMC or CasII-gly with vitamin E analogues in HeLa cell two-dimensional cultures.

Drug 1	*assayed doses (µM)*	*Drug 2*	*assayed doses (µM)*	*C values (BTA %) [Range]*	*Experimental values % [Range]*	*Synergism (%) [Range]*
Celecoxib	5-10	α-TOS	0.1-1	26 ± 0.8 [25-27]	19 ± 6 [13-23]	-(7 ± 5) [-(4 -13)]
	10-20	α-TEA	0.1-4	11 ± 9.5 [4-22]	23.5 ± 6 [17-28]	12 ± 10 [3-23.5]
DMC	25-30	α-TOS	0.1-1	42 ± 1.5 [40.5-43.5]	28 ± 2 [26-30]	-(14 ± 2) [-(13-16)]
	30-25	α-TEA	0.1-4	30 ± 6 [26-37]	-(37 ± 5.5) [-(33-43)]	- (66.5 ± 11) [-(60– 80)]
CasII-gly	0.3 – 0.5	α-TOS	0.1 – 1	25 ± 14 [8.5-33]	7 ± 3 [4.5-11]	- (18 ± 13) [-(3 – 28)]
	0.3 – 0.5	α-TEA	0.5 – 4	33± 6 [29-39.5]	16 ± 3 [12-19]	- (17 ± 4) [-(13 – 20)]
	0.3 – 0.5	M-TEA	0.1-1	36± 1.5 [34-37]	19.5 ± 7 [14-28]	-(16 ± 8) [-(7-23)]

Infra-additive effects of celecoxib, DMC or CasII-gly with vitamin E analogues in HeLa MCTS cultures

			Preventive protocol			
Drug 1	*assayed doses (nM)*	*Drug 2*	*assayed doses (nM)*	*C values (BTA %) [Range]*	*Experimental values % [Range]*	*Synergism (%) [Range]*
Celecoxib	0.1-0.5	α-TOS	1-10	37 ± 5 [33-43]	19 ± 6 [13-23]	-(18 ± 10) [-(11 – 30)]
	0.5-1	α-TEA	15-25	44 ± 8 [38-53]	23.5 ± 6 [17-28]	-(21 ± 13) [-(12 – 36)]

DMC	1-10	α-TOS	1-10	45 ± 2 [43.5-48]	23 ± 6 [17-29]	-(22 ± 6) [-(15 – 26)]
	5-10	α-TEA	15-25	35 ± 12 [22-47]	19.5 ± 3 [17-22]	-(15 ± 15) [-(0.6 –29)]
CasII-gly	10-30	α-TOS	1-10	26 ± 15 [8.5-35]	16 ± 8 [7-22]	-(10 ± 7) [-(1 - 13)]
	1-5	α-TEA	15-25	35 ± 5 [30-40]	16 ± 4 [12-19]	-(18 ± 8) [-(12 – 28)]

Curative Protocol

Drug 1	*assayed doses (μM)*	*Drug 2*	*assayed doses (μM)*	*C values (BTA %) [Range]*	*Experimental values % [Range]*	*Synergism (%) [Range]*
Celecoxib	1-5	α-TOS	10-50	26 ± 0.7 [25-27]	19 ± 6 [13-23]	-(7 ± 5) [-(3 - 13)]
	3-7	α-TEA	25-30	39 ± 7 [34-47]	23.5 ± 6 [17-28]	- (15 ± 13) [-(6 – 30)]
DMC	10-25	α-TOS	10-50	46 ± 7[39-52]	28 ± 2 [26-30]	-(18 ± 8) [-(9 - 25)]
	25-35	α-TEA	25-30	29 ± 5 [26-34.5]	22 ± 4 [17-25]	-(7 ± 9) [-(1 - 17)]
CasII-gly	10-50	α-TOS	10-50	29 ± 7 [20-34]	7 ± 3 [4.5-11]	-(22 ± 6) [-(15 - 28)]
	100-200	α-TEA	24-30	33 ± 3[29-35.5]	16 ± 4 [12-19]	-(16 ± 7) [-(10 - 23)]

For HeLa bidimensional cultures, the IC_{50}values of α-TOS, α-TEA and M-TEA, as single agents, were 1.2 ± 0.46 μM, 4.7 ± 0.7 μM, and 1.2 ± 0.48 μM, respectively. For the MCTS preventive protocol, the IC_{50}values of α-TOS and α-TEA were 10 ± 4 nM and 25 ± 6 nM, respectively. For the MCTS curative protocol, the IC_{50}values of α-TOS and α-TEA were 50 ± 11 μM and 30 ± 2 μM, respectively.
Abbreviations: α-TOS, α-tocopheryl succinate; α-TEA, α-tocopherol ether linked acetic acid analog; M-TEA,methoxy-tocopheryloxyacetic acid. The Bliss-Type Additivism (BTA) was calculated as outlined in the Material and Methods section.

Table S2

Synergistic effects of NSAIDs with canonical anti-cancer drugs at sub-IC50 concentrations in bidimensional HeLa cell cultures as shown by using the Combination Index (CI) value

Drug 1	*assayed doses (μM)*	*Drug 2*	*assayed doses (μM)*	*CI [Range]*
Celecoxib	5-10	Cisplatin	2-5	0.2 ± 0.10 [0.1-0.3]
	5-10	Paclitaxel	11-15	0.65 ± 0.2 [0.4-0.9]
	5-10	Doxorubicin	10-20	0.7 ± 0.1 [0.5-0.8]
DMC	10-15	Cisplatin	2-5	0.2 ± 0.01 [0.19-0.22]
	20-25	Paclitaxel	20-21	0.8 ± 0.09 [0.7-0.9]
	20-25	Doxorubicin	10-20	0.9 ± 0.1 [0.8-1]
CasII-gly	0.5-1	Cisplatin	50-100	0.7 ± 0.2 [0.5-0.9]
	0.5-1	Paclitaxel	10-20	0.55 ± 0.2 [0.35-0.75]
	0.5-1	Doxorubicin	10-20	0.9 ± 0.02 [0.85-0.9]

CI was calculated as outlined in the Material and Methods section. The data shown represent the mean ± S.D. of at least three different independent bidimensional cultures (n=3).

Figure S1

HeLa bidimensional

% Inhibition of growth

	0	1	2.5	5	10
20	84	-2	0	98	94
15	50	9	-20	85	87
10	43	-14	-5	77	74
5	38	-3	5	46	50
1	15	-3	-3	22	35
0	0	0	0	2	6

% BTA

Cisplatin (µM) \ Celecoxib (µM)	0	1	2.5	5	10
20	0	-82	-84	12	10
15	0	-41	-30	33	31
10	0	-98	-38	32	25
5	0	-41	-33	21	6
1	0	-18	-18	5	14
0	0	0	0	0	0

MCTS

Preventive Protocol

% Inhibition of growth

Cisplatin (nM) \ Celecoxib (nM)	0	0.1	0.4	0.7	1
80	85	-2	98	20	94
75	75	-4	98	35	87
50	74	-3	71	45	74
25	55	-7	68	10	50
10	50	-45	47	75	35
0	0	0	2	1.5	4

% BTA

Cisplatin (nM) \ Celecoxib (nM)	0	0.1	0.4	0.7	1
80	0	-87	11	-66	5
75	0	-79	21	-41	8
50	0	-77	19	-30	20
25	0	-62	41	-46	-9
10	0	-95	35	23	-20
0	0	0	0	0	0

Curative Protocol

% Inhibition of growth

Cisplatin (µM) \ Celecoxib (µ M)	0	2	4	8
300	80	99	-5	100
200	74	75	-2	99
100	64	65	-3	94
10	45	36	0	99
5	10	15	-1	98
0	0	4	5	7

% BTA

Cisplatin (µM) \ Celecoxib (µM)	0	2	4	8
300	0	15	-90	13
200	0	-3	-51	18
100	0	-14	-71	23
10	0	-13	-50	82
5	0	1	-16	86
0	0	0	0	0

FigureS1. Representative drug matrixes showing the effect of cisplatin *plus* celecoxib on HeLa cell cultures. Upper panel: the percentages of cell proliferation inhibition from single drug used are shown in white boxes; for drug combinations, these are shown in gray boxes. Lower panel: Bliss-type Additivism (BTA) percentage values, light gray boxes indicate an infra-additive effect; dark gray boxes indicate a supra-additive effect.

Figure S2

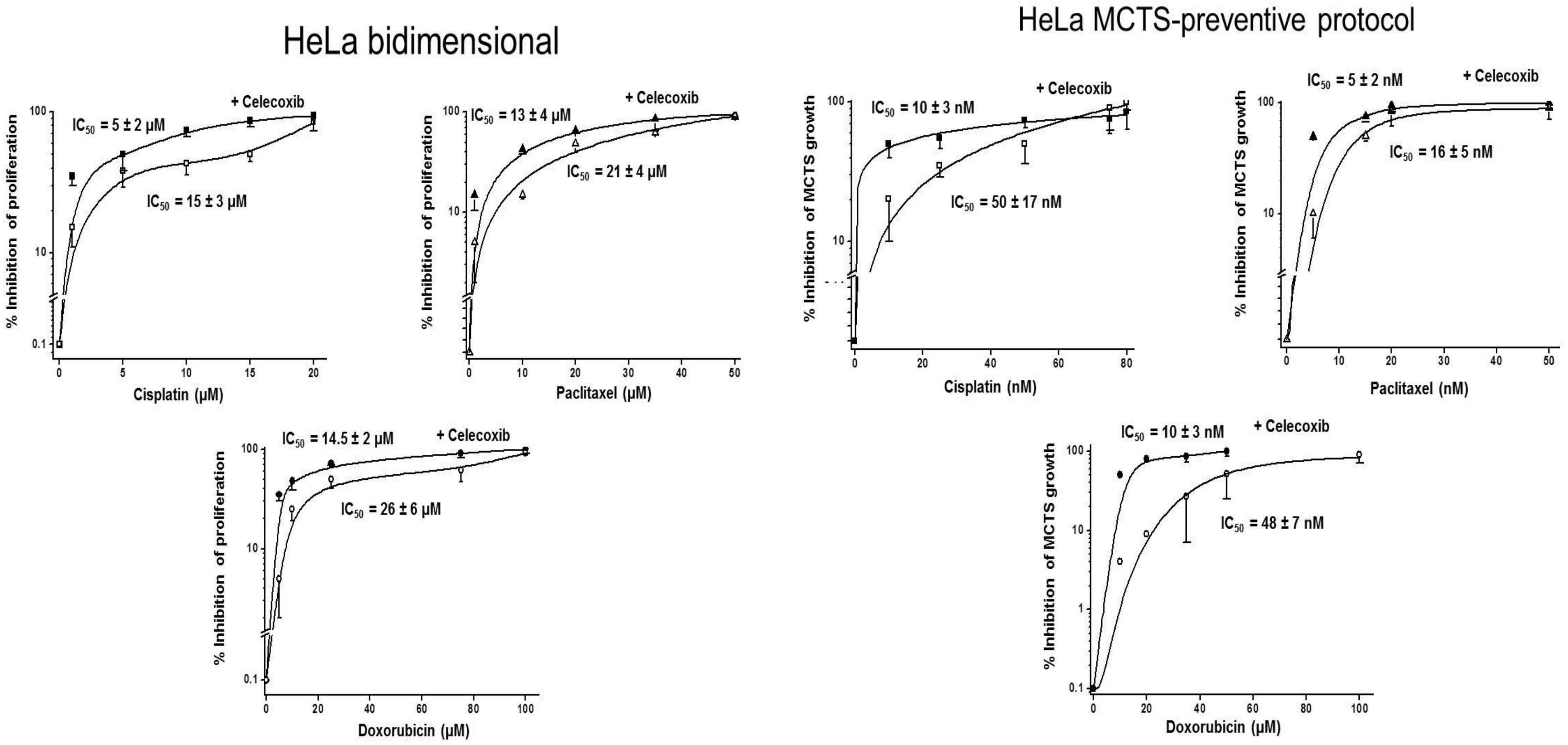
HeLa bidimensional
HeLa MCTS-preventive protocol
+ Celecoxib
IC50 = 5 ± 2 μM
IC50 = 15 ± 3 μM
Cisplatin (μM)
% Inhibition of proliferation
IC50 = 13 ± 4 μM
IC50 = 21 ± 4 μM
Paclitaxel (μM)
IC50 = 14.5 ± 2 μM
IC50 = 26 ± 6 μM
Doxorubicin (μM)
IC50 = 10 ± 3 nM
IC50 = 50 ± 17 nM
Cisplatin (nM)
% Inhibition of MCTS growth
IC50 = 5 ± 2 nM
IC50 = 16 ± 5 nM
Paclitaxel (nM)
IC50 = 10 ± 3 nM
IC50 = 48 ± 7 nM
Doxorubicin (μM)

HeLa MCTS-curative protocol

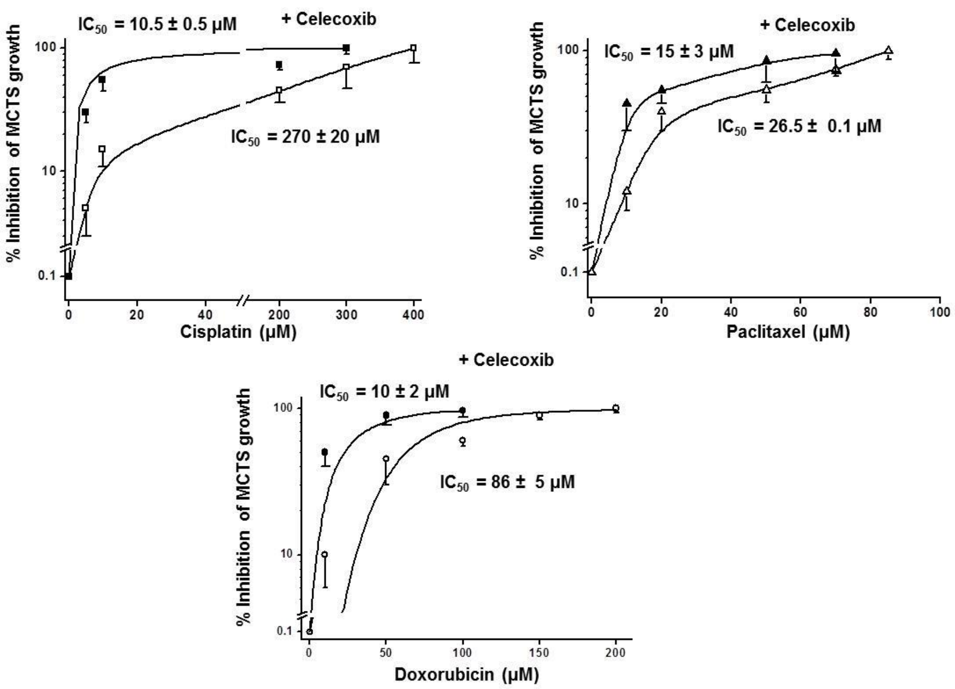

Figure S2. Logarithmic dose-response curve shows the effect of cisplatin, paclitaxel and doxorubicin on HeLa bidimensional cultures (n=3) and MCTS under preventive and curative protocols (n= 30 MCTS), with the presence of celecoxib. For bidimensional cultures, celecoxib was added at 5 -10 µM. For MCTS, celecoxib was added at 0.4-1 nM or 2-6 µM in preventive or curative protocols, respectively.

Figure S3

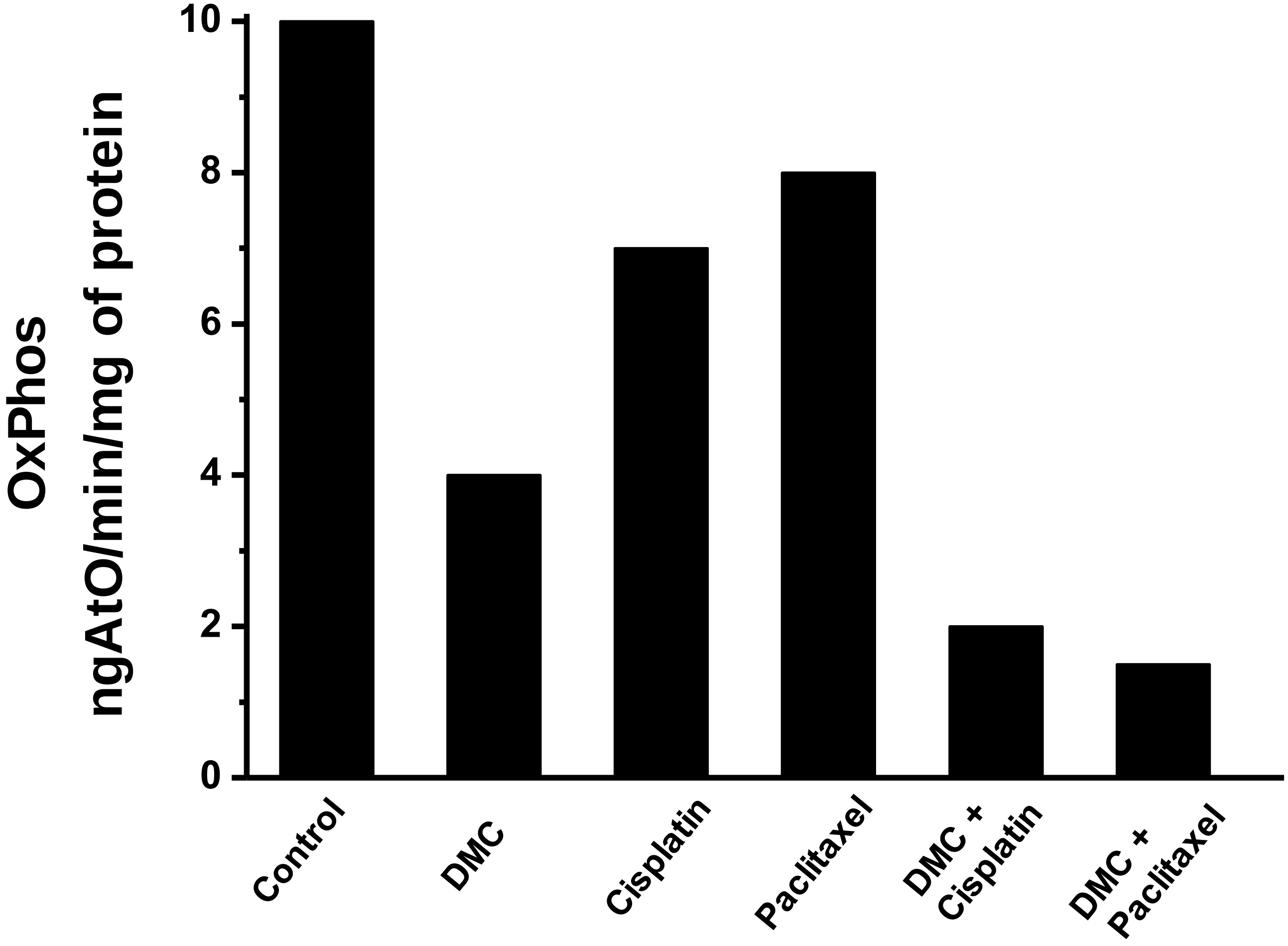

Figure S3. Effect of DMC (15 µM), paclitaxel (20 µM) and cisplatin (5 µM) alone or in combination, on OxPhos fluxes, after 24 h exposure in HeLa cells. n=1; control (no added drugs).

Figure S4

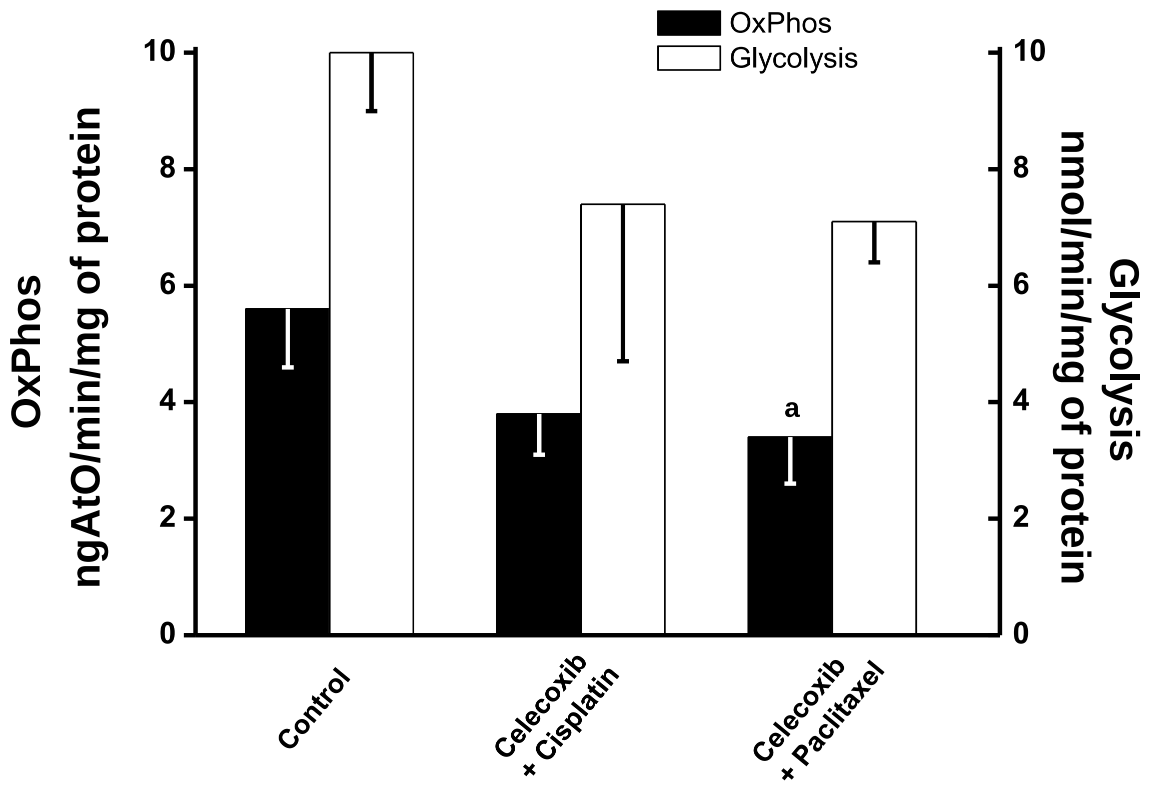

Figure S4. Effect of celecoxib (5 μM), paclitaxel (15 μM) and cisplatin (2 μM) added in combination on OxPhos and glycolysis fluxes, after 24 h exposure in mouse 3T3 fibroblast. The data show the mean ± S.D. of at least three different preparations. [a]P< 0.05 *vs.* control (no added drugs).

FIGURE S5.

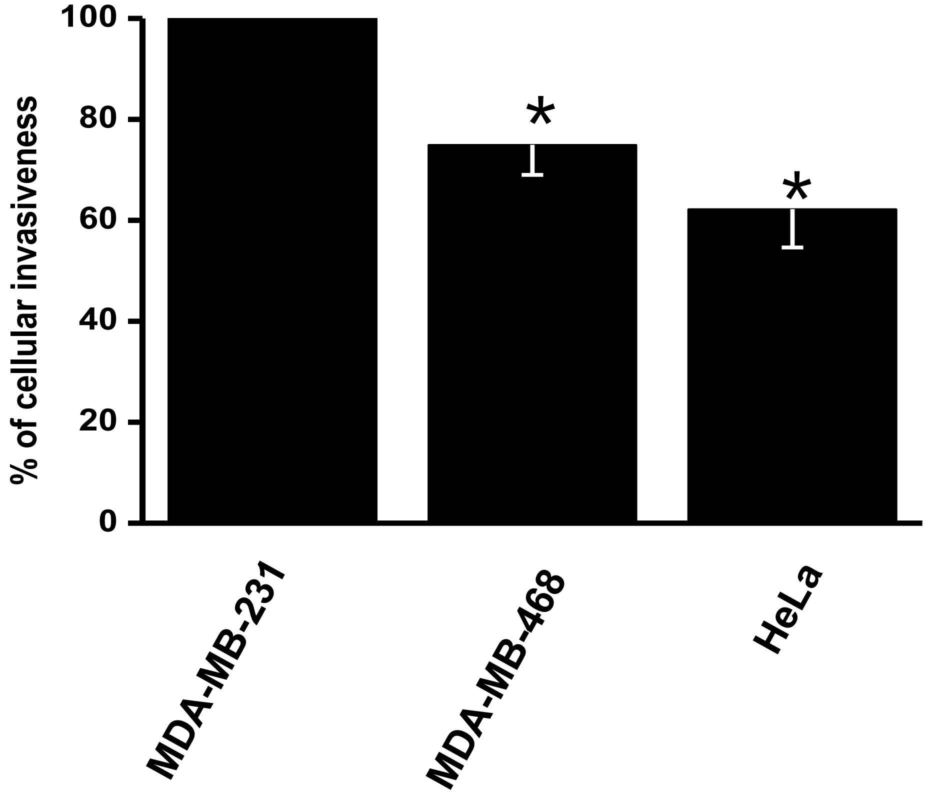

Figure S5. Invasiveness potential of different metastatic cancer cell lines. n= 3, *P<0.05 *vs.* MDA-MB-231.

Figure S6

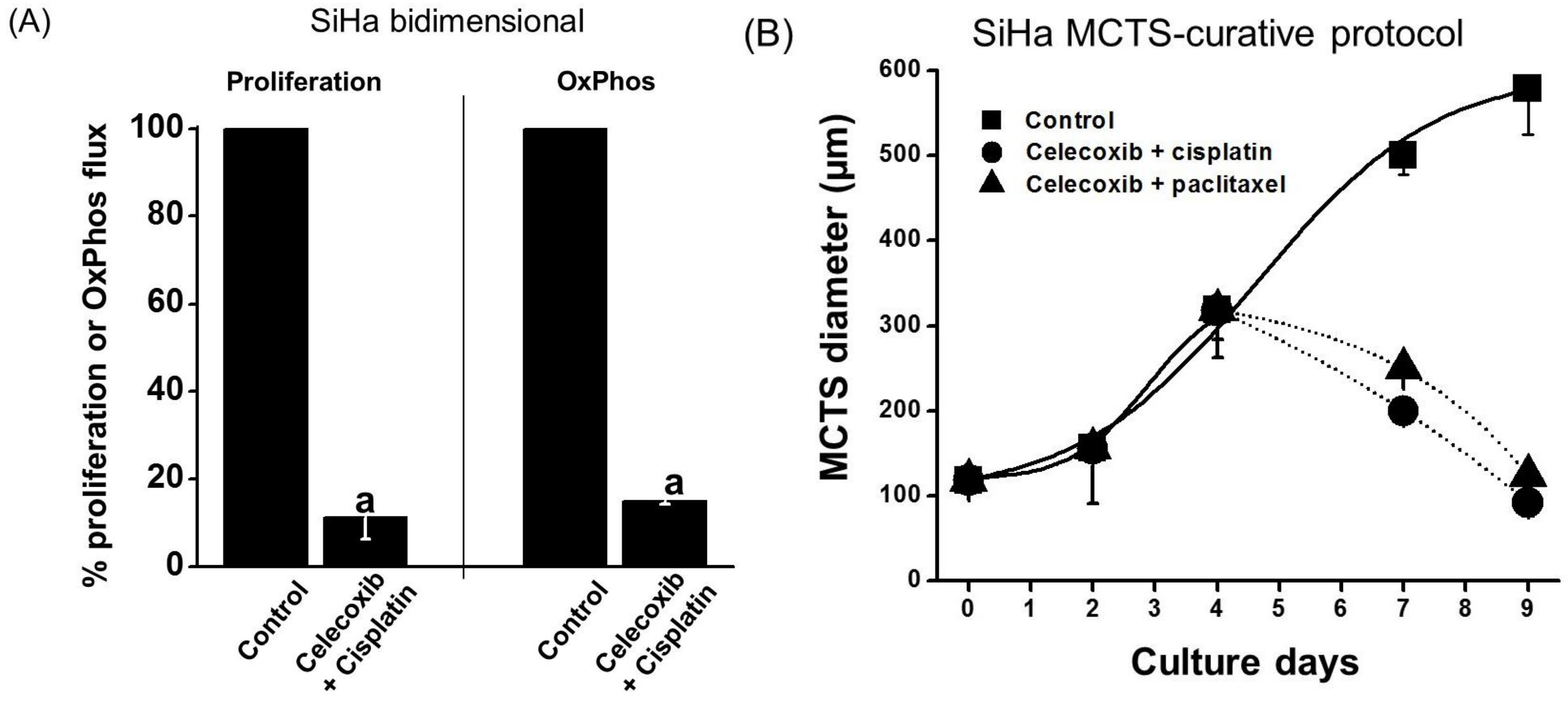

Figure S6. (A) Effect of celecoxib (3 µM) and cisplatin (1 µM) added in combination on cell proliferation and OxPhos flux, after 24 h exposure in SiHa bidimensional cultures. Control cell proliferation (100%) corresponded to 34 x 10^3 cells after 48 h culture. OxPhos flux control (100%) corresponded to 10 ± 1.5 ng At O/min/mg of protein. The data shown represent the mean ± S.D. of at least three different preparations for bidimensional culture. [a]P < 0.05 *vs.* control (no added drugs).
(B) Effect of celecoxib (2 µM), cisplatin (1 µM) and paclitaxel (10 µM) added in combination on SiHa MCTS growth using curative protocol (n= 5 spheroids)

Figure S7

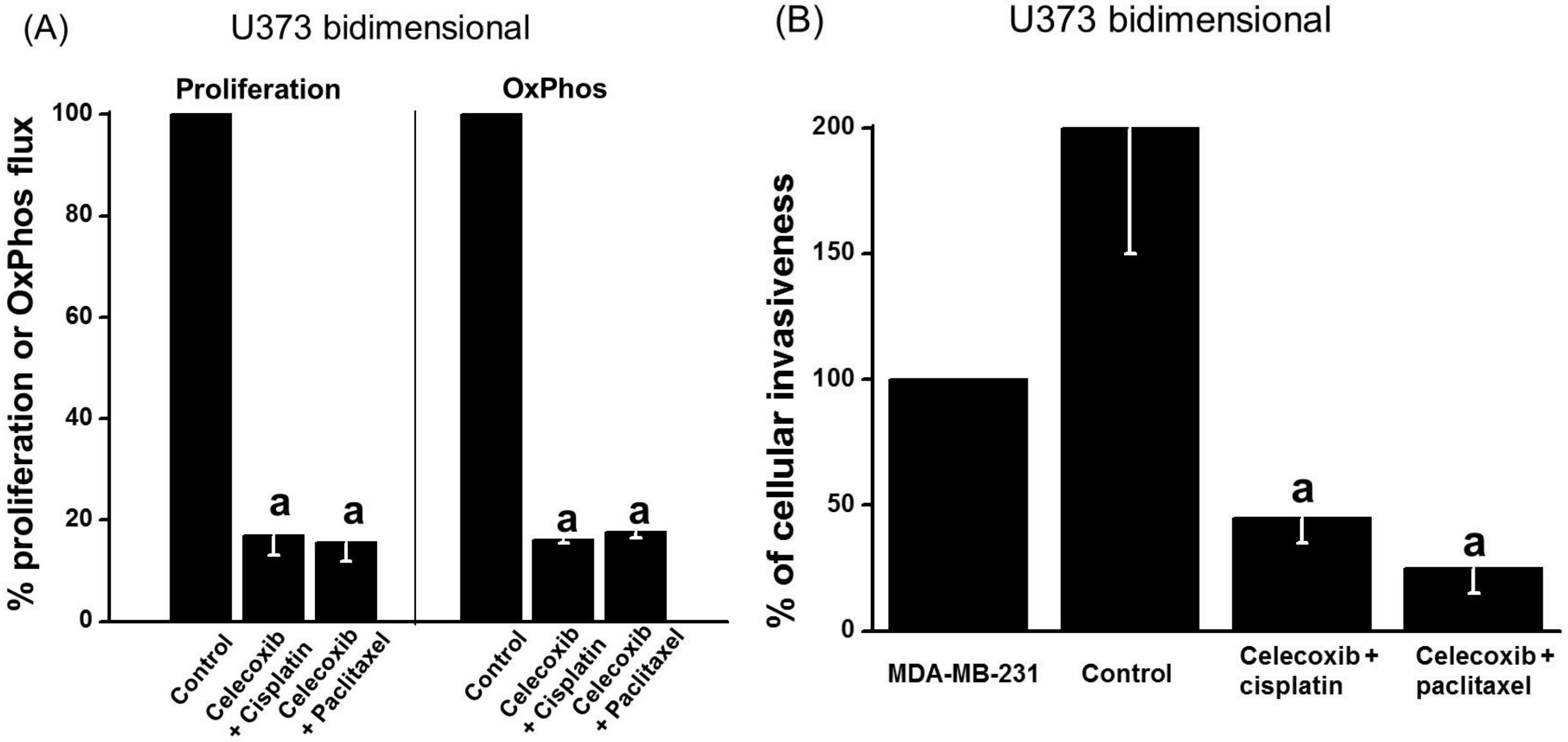
(A)
U373 bidimensional
Proliferation
OxPhos
% proliferation or OxPhos flux
100
80
60
40
20
0
a
a
a
a
Control
Celecoxib + Cisplatin
Celecoxib + Paclitaxel
Control
Celecoxib + Cisplatin
Celecoxib + Paclitaxel
(B)
U373 bidimensional
% of cellular invasiveness
200
150
100
50
0
a
a
MDA-MB-231
Control
Celecoxib + cisplatin
Celecoxib + paclitaxel

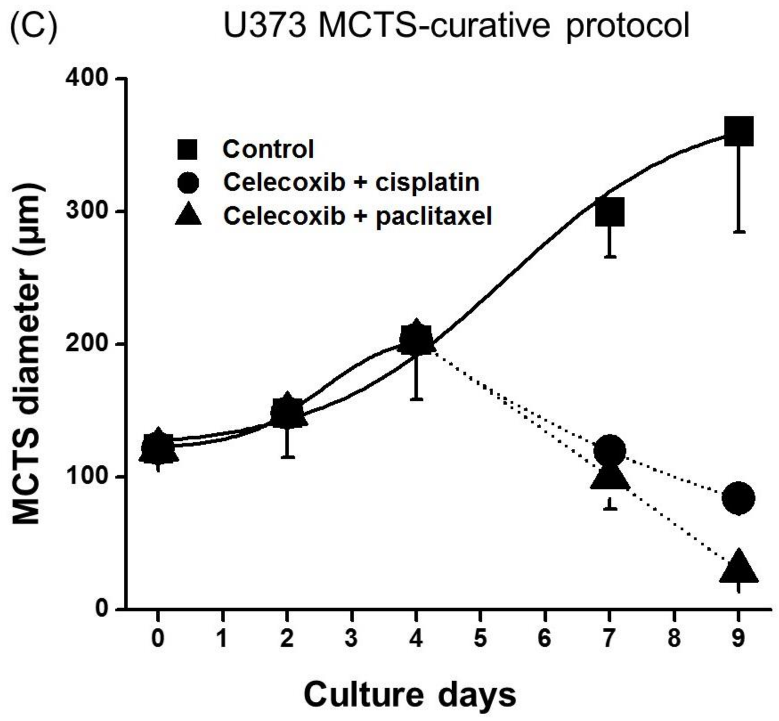

Fig S7. Effect of celecoxib (5 μM), cisplatin (2 μM) and paclitaxel (15 μM) added in combination on (A) cell proliferation, OxPhos flux and (B) invasiveness after 24 h exposure in U373 bidimensional cultures. Control cell proliferation (100%) corresponded to 48 x 10^3 cells. OxPhos flux control (100%) corresponded to 13 ± 5 ng At O/min/mg of protein. The data show the mean ± S.D. of at least three different preparations for bidimensional culture. $^{a}P < 0.05$ *vs.* control (no added drugs). (C) Effect of celecoxib (4-5 μM), cisplatin (1 μM) and paclitaxel (25 μM) added in combination on U373 MCTS growth using curative protocol (n= 5 spheroids).
